# Adhesion‐Enhanced Engineered Probiotic for Prolonged Intestinal Retention and Calprotectin‐Responsive IBD Therapy

**DOI:** 10.1002/advs.77743

**Published:** 2026-09-11

**Authors:** Yuxi Wang, Peichao Zhang, Simin Gu, Hongyun Lu, Ningning Jiang, Jiarun Zhao, Enbo Xu, Xiayu Liu, Ying Shi, Qihe Chen

**Affiliations:** ^1^ Department of Food Science and Nutrition Zhejiang University Hangzhou China; ^2^ Future Food Laboratory Innovation Centre of Yangtze River Delta Zhejiang University Jiaxing China; ^3^ School of Life and Health Sciences Hubei University of Technology Wuhan China; ^4^ Zhejiang Key Laboratory of Functional Structural Lipid Synthesis and Application Jiashan China

**Keywords:** antioxidant enzyme systems, calprotectin‐responsive circuits, engineered probiotics, inflammatory bowel disease, intestinal retention, live biotherapeutic products, mucosal adhesion

## Abstract

The clinical translation of engineered probiotics for inflammatory bowel disease (IBD) is hindered by limited intestinal retention and insufficient inflammation‐responsive precision. This study utilizes a surface display strategy to construct an adhesion‐enhanced *Escherichia coli* Nissle 1917 chassis. By presenting a truncated SpaC adhesin derived from *Lactobacillus rhamnosus* GG via the ice nucleation protein system, the engineered strain achieves enhanced mucosal anchoring and prolonged intestinal retention. To achieve an autonomous response, the probiotic platform integrates a calprotectin‐responsive *ykgMO* promoter to drive a CAT‐SOD‐GPx tripartite fusion system (TFS) and secretion of human trefoil factor 3 (TFF3). In the TFS, CAT and SOD mediate ROS scavenging, whereas GPx is proposed to serve as a non‐catalytic structural‐support component associated with improved folding and soluble expression. Combined with pH‐responsive Eudragit L100‐55 microencapsulation to ensure gastric survival, this programmable system effectively alleviates inflammation, restores epithelial barrier integrity, and reverses oxidative damage in both prophylactic and therapeutic murine colitis models. Furthermore, the platform remodels the gut microbiota by suppressing opportunistic pathobionts such as *Escherichia‐Shigella* while simultaneously promoting the resurgence of beneficial taxa, including *Lachnospiraceae*. Together, this biomarker‐responsive strategy offers a versatile and programmable framework for the precision management of IBD during acute flare‐ups.

## Graphical abstract

An intelligent engineered probiotic platform is developed for the precision management of inflammatory bowel disease. By integrating surface‐displayed adhesins with an inflammation‐responsive CAT‐SOD‐GPx tripartite fusion system and mucosal repair factors, this programmable system achieves prolonged intestinal retention and multi‐pronged therapy. It effectively scavenges reactive oxygen species (ROS), promotes mucosal healing, and restores microbiota homeostasis in acute colonic inflammation.

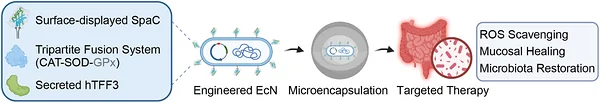

## 1 Introduction

Inflammatory bowel disease (IBD), primarily comprising ulcerative colitis and Crohn's disease, is a chronic, relapsing inflammatory disorder of the gastrointestinal tract affecting millions of individuals worldwide [1, 2]. Despite advances in clinical interventions, including aminosalicylates, corticosteroids, and monoclonal antibodies targeting tumour necrosis factor (TNF), a definitive cure remains elusive. Most current pharmacological strategies rely on systemic administration, often necessitating high dosages to achieve therapeutic concentrations in the distal colon, which frequently results in significant systemic toxicity and immunosuppression [3, 4]. Furthermore, these broad‐spectrum anti‐inflammatory agents inadequately address the underlying gut microbial dysbiosis, a hallmark of IBD that exacerbates mucosal injury and perpetuates chronic inflammation [5, 6]. Thus, there is an imperative demand for site‐specific therapeutic platforms capable of simultaneously alleviating inflammation and restoring microbial homeostasis.

The engineering of probiotics as Live Biotherapeutic Products (LBPs) offers a promising localized strategy for IBD management [7, 8, 9, 10]. Among various candidates, *Escherichia coli* Nissle 1917 (EcN) is a preferred clinical chassis due to its established safety profile and versatile genetic toolkits [11, 12]. Nevertheless, the efficacy of EcN is fundamentally limited by poor intestinal persistence, as diarrheal washout and colonization resistance from resident microbiota cause rapid clearance of exogenous strains. While material‐based delivery systems improve initial colonic delivery, these physical barriers are transient and inevitably degrade during bacterial proliferation [13, 14]. Consequently, the unprotected progeny lack competitive retention advantages, leading to a narrow therapeutic window. Thus, developing a genetically encoded strategy for stable, inheritable adhesion is essential for long‐term therapeutic efficacy.

To identify an optimal molecular anchor, we focused on the exceptional mucosal adhesion traits of *Lactobacillus rhamnosus* GG (LGG), a probiotic strain renowned for its robust persistence in the human gut [15, 16]. The intestinal retention of LGG is primarily mediated by SpaC, a critical adhesin localized at the pilus tip. SpaC possesses a high affinity for both intestinal mucins and extracellular matrix (ECM) proteins, components often exposed in ulcerated lesions of IBD patients [17, 18]. Crucially, SpaC‐mediated binding exhibits remarkable resistance to gastrointestinal shear stress, making its functional domain a powerful tool for enhancing the biointerfacial anchoring of engineered microbes.

In this work, we designed and constructed an engineered probiotic platform with genetically inherited mucosal adhesion. We employed the ice nucleation protein surface display system to present the adhesion domain of SpaC on the EcN outer membrane. Unlike temporary physical encapsulation, this genetically integrated strategy ensures that the adhesion‐enhanced phenotype is faithfully transmitted to subsequent generations. By establishing multiscale interactions with intestinal mucins and epithelial cells, this engineered chassis can bypass colonization resistance and withstand luminal washout, thereby creating a robust, high‐density niche within the inflamed colon for sustained therapeutic production.

**FIGURE 1:**
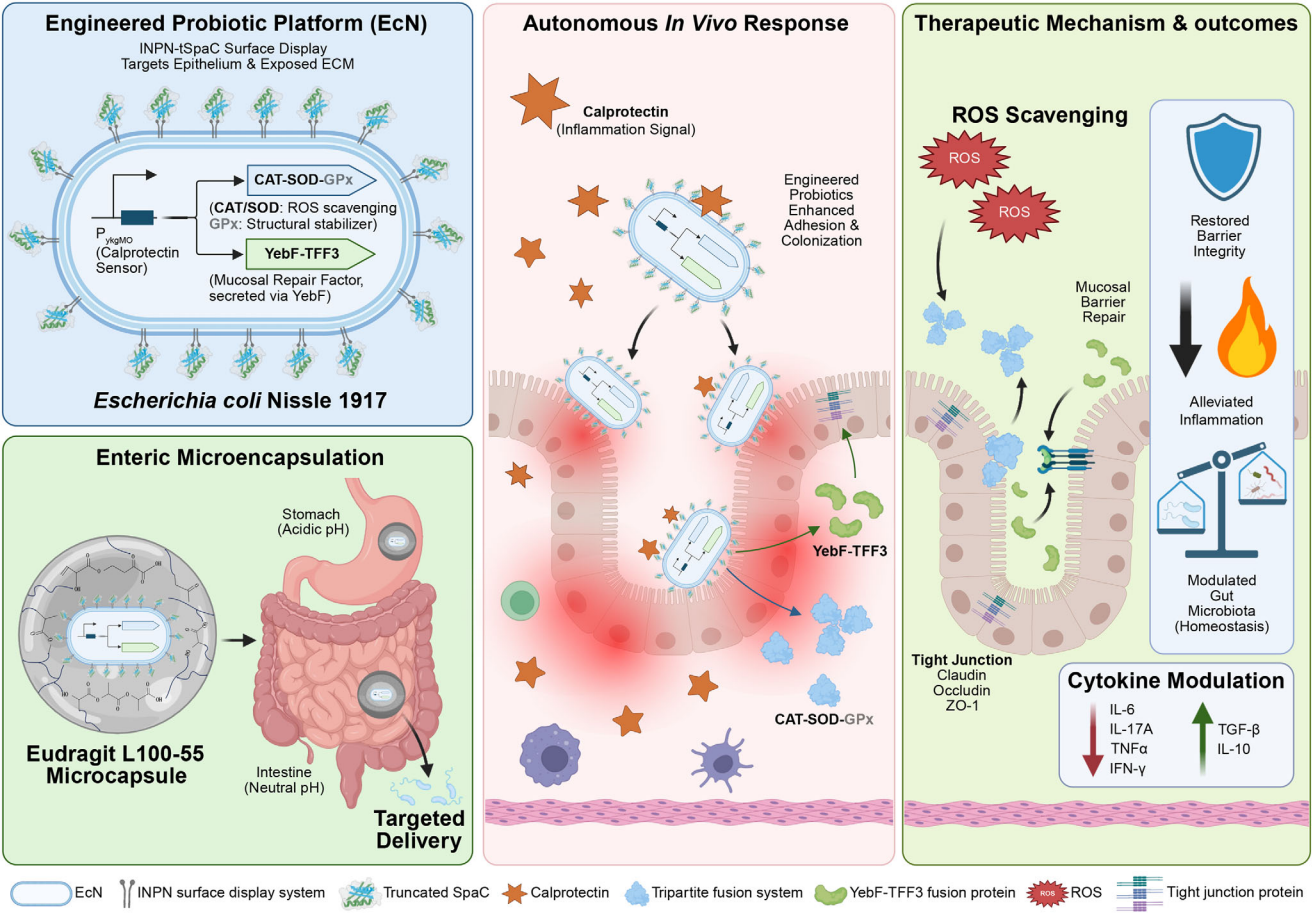
Design, delivery, and therapeutic mechanism of the programmable EB4@L100‐55 probiotic platform. The EcN chassis is engineered for the surface display of truncated SpaC (tSpaC) via the ice nucleation protein N‐terminus (INPN) system. The inflammation‐responsive therapeutic system is driven by a calprotectin‐responsive *ykgMO* promoter and consists of a CAT‐SOD‐GPx tripartite fusion system (TFS) and a human trefoil factor 3 (TFF3) secretion module. To safeguard bacterial viability during gastric transit and ensure efficient intestinal delivery, the engineered EcN are encapsulated within pH‐responsive Eudragit L100‐55 microcapsules. The sensing of pathological calprotectin at inflamed sites triggers the localized release of therapeutic payloads for ROS scavenging and mucosal repair. Biological outcomes depicted include the modulation of tight junction proteins (Claudin, Occludin, and ZO‐1), the rebalancing of systemic cytokines (IL‐6, IL‐17A, TNF‐α, IFN‐γ, TGF‐β, and IL‐10), and the restoration of gut microbial homeostasis. Created with BioRender.com.

On the basis of this retention‐enhanced chassis, we further integrated synthetic biology modules to enable autonomous, biomarker‐responsive therapy. Specifically, we engineered the adhesion‐enhanced EcN to harbour a genetic circuit under the control of the calprotectin‐responsive *ykgMO* promoter, enabling sensing of this clinically established IBD activity marker [19, 20]. Upon activation, the system drives coordinated expression of a CAT‐SOD‐GPx tripartite fusion system (TFS) and the secretion of trefoil factor 3 (TFF3). Within the TFS, CAT and SOD mediate reactive oxygen species (ROS) scavenging, whereas GPx may contribute to the structural stabilization and soluble expression of the fusion construct. To ensure gastric survival, the engineered EcN were further protected via enteric microencapsulation. Through extensive validation in both prophylactic and therapeutic murine models of colitis, we demonstrate that this programmable, adhesion‐enhanced probiotic platform confers robust protection against inflammation, maintains gut barrier integrity, and restores microbial diversity. These findings establish a promising strategy for the precision treatment of IBD, particularly during acute flare‐ups (Figure 1).

## 2 Results

### 2.1 Construction and Characterization of Adhesion‐Enhanced EcN via SpaC Surface Display

**FIGURE 2:**
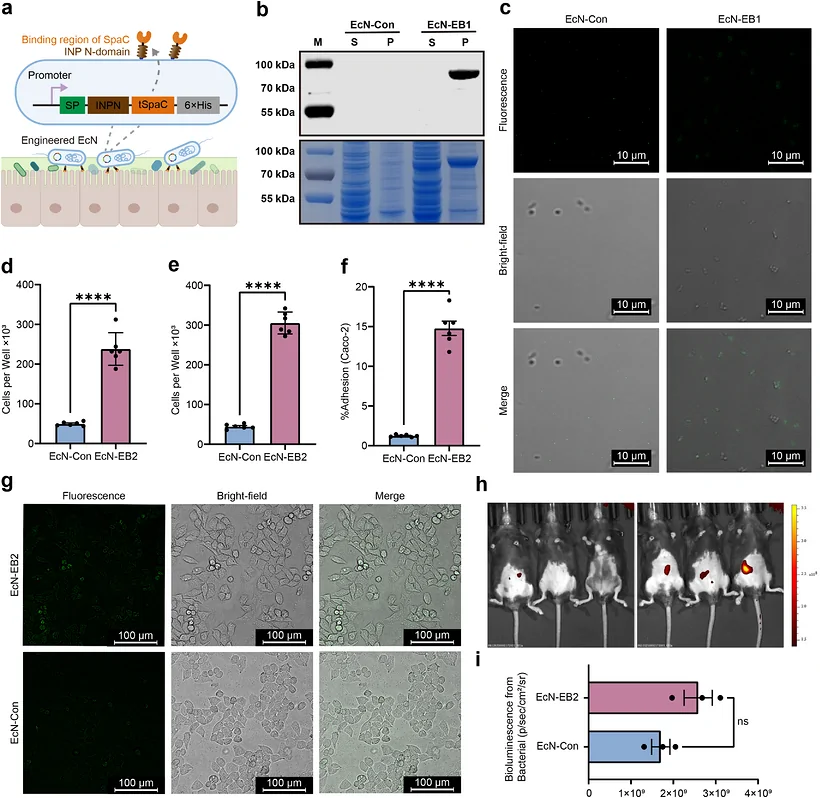
Construction and functional characterization of the tSpaC surface display system in EcN. (a) Schematic illustration of the truncated SpaC (tSpaC) surface display system utilizing the INPN anchor and its interaction with the intestinal epithelium. (b) Characterization of tSpaC expression in the EcN‐EB1 strain via SDS‐PAGE (bottom) and Western blot (top). M, S, and P denote the protein marker, supernatant, and pellet fractions of cell lysates, respectively. (c) Confocal laser scanning microscopy (CLSM) images of EcN‐EB1 following live‐cell immunofluorescence staining (green). Scale bar, 10 µm. (d–f) In vitro adhesion capacity of EcN‐EB2 to mucin (d), Type IV collagen (e), and Caco‐2 cell monolayers (f), quantified by plate colony counting. (g) Representative inverted fluorescence microscopy images showing EcN‐EB2 (green) adhering to Caco‐2 monolayers. Scale bar, 100 µm. (h, i) In vivo intestinal retention kinetics in healthy mice. Representative bioluminescence images (h) and statistical analysis of fluorescence intensity (i) are shown. Data are presented as mean ± SEM (*n* = 6 independent biological replicates for d–f; *n* = 3 biologically independent animals for i). Statistical significance was determined using a two‐tailed Student's t‐test (^****^ *p* < 0.0001; ns, not significant).

To overcome the limited gastrointestinal retention of exogenous microbes, we sought to engineer EcN to surface‐display SpaC, a multifunctional pilin adhesin from LGG with broad‐spectrum mucosal affinity (Figure 2a). We initially performed systematic screening of promoter strengths and surface display architectures using the pET‐22b(+) backbone. Two constitutive promoters of varying intensities (low‐strength BBa_J23114 and high‐strength BBa_J23119) were screened in combination with three established bacterial scaffolds: the ClyA system [21, 22], the N‐terminal domain of the ice nucleation protein (INPN) system from *Pseudomonas syringae* [23], and the AIDA system [24], with a flexible GGGGSGS linker tethering the scaffolds to SpaC. However, repeated attempts to clone the *spaC* gene into constitutive expression platforms consistently failed. Transformation yielded negligible recombinant colonies, and sequencing of the rare survivors revealed the ubiquitous presence of inactivating mutations, characterized predominantly by frameshifts and large‐scale deletions within the SpaC encoding sequence. These results reflect a potent negative selection pressure driven by the substantial metabolic burden and potential cytotoxicity associated with SpaC expression, suggesting that constitutive production exceeded the physiological tolerance of EcN and triggered rapid genetic instability.

To circumvent this instability, we transitioned to a more tightly regulated, L‐(+)‐arabinose‐inducible araBAD system. A 1231‐bp regulatory cassette encoding the araC repressor, araBAD promoter, and ribosome binding site (RBS) was subcloned from the pBAD vector to replace the constitutive promoters. However, SpaC expression remained undetectable by SDS‐PAGE. Given that surface display efficiency is often limited by passenger protein size, we hypothesized that the full‐length SpaC (89.0 kDa) exceeded this threshold. To address this, we engineered a truncated SpaC variant (tSpaC, residues T36–V500, 50.6 kDa) retaining only the core N‐terminal binding domain to alleviate the metabolic burden on protein folding and translocation [18]. A comparative screening across three candidate display systems revealed that the target protein was exclusively detectable in the insoluble pellet fraction of the INPN system. Through this iterative optimization, the INPN scaffold was identified as the only system capable of successfully anchoring tSpaC to the outer membrane (Figure S1).

To further minimize inclusion body formation and enhance translation fidelity, we designed a codon‐optimized INPN‐tSpaC cassette integrated into the pET‐22b(+) vector downstream of the pelB signal peptide. The tSpaC sequence was fused to the INPN scaffold via a flexible (GGGGSGS) linker, with a C‐terminal 6 × His tag for detection. The native T7 promoter within the pET‐22b(+) vector was subsequently substituted with the L‐(+)‐arabinose‐inducible araBAD promoter, yielding the plasmid pET‐araBAD‐tSpaC, which was then transformed into EcN to establish the engineered strain EcN‐EB1. Robust expression of the INPN‐tSpaC fusion protein was confirmed by SDS‐PAGE and Western blot analyses, revealing a prominent band situated between 70 and 100 kDa markers, consistent with its predicted molecular weight of 74.4 kDa (Figure 2b). Notably, the fusion protein was predominantly detected in the insoluble fraction of the cell lysate. To distinguish whether this localization represented legitimate membrane anchoring or the formation of misfolded inclusion bodies, whole‐cell immunofluorescence staining was performed on intact bacteria. Confocal microscopy revealed robust colocalization of the fluorescence signal with the EcN‐EB1 cell surface, whereas wild‐type EcN exhibited negligible background (Figure 2c). Collectively, these data confirm the proper folding and spatial anchoring of INPN‐tSpaC as a functional surface‐displayed adhesin. We further assessed the physiological impact of tSpaC display on the probiotic chassis through growth kinetic analysis. In the absence of the inducer L‐(+)‐arabinose, the growth profile of EcN‐EB2 was nearly identical to that of the control strain EcN‐Con, suggesting that the regulated system maintains minimal metabolic leakage under repressed conditions. Conversely, addition of L‐(+)‐arabinose triggered the expression of tSpaC and led to a discernible inhibition of the bacterial growth rate (Figure S2). This observation further corroborates the significant metabolic cost associated with translocating and anchoring of complex adhesins, underscoring the necessity of an inducible system to balance bacterial fitness with therapeutic functionality.

### 2.2 Enhanced Adhesion and Intestinal Retention via Surface‐Displayed tSpaC

To address the exposure of the epithelium and ECM caused by the compromised mucus barrier in inflammatory bowel disease [25, 26, 27], we investigated whether surface displayed tSpaC maintains sufficient anchoring capability. Although native SpaC is known for its broad‐spectrum adhesive affinity toward various host substrates, whether the truncated tSpaC, when displayed via the INPN scaffold, retains sufficient anchoring capability to these target surfaces remains to be elucidated. Thus, we systematically evaluated the adhesive performance of surface‐displayed tSpaC using both in vitro and in vivo models. Prior to these assessments, a fluorescent reporter system was developed to facilitate high‐sensitivity tracking and quantification. Specifically, a constitutive expression cassette comprising the strong BBa_J23119 promoter and mNeonGreen [28] (hereafter mGFP) was integrated into the pET‐22b(+) and pET‐araBAD‐tSpaC vectors, generating the control strain EcN‐Con and the adhesion‐enhanced platform strain EcN‐EB2, respectively.

We first quantified the adhesive profile of EcN‐EB2 across three physiologically relevant in vitro models: mucin, collagen IV (a primary basement membrane component), and Caco‐2 intestinal epithelial cell monolayers. For protein‐binding assays, mucin and collagen IV were immobilized on Nunc MaxiSorp flat‐bottom 96‐well plates, while Caco‐2 cells were cultured in 6‐well plates to form confluent monolayers. Following the incubation of EcN‐Con or EcN‐EB2 within these substrates, quantitative analysis of the adherent bacteria revealed a profound enhancement in the binding capacity of EcN‐EB2. Specifically, EcN‐EB2 exhibited a 4.79‐fold and 6.86‐fold increase in adhesion to mucin and collagen IV, respectively, compared to EcN‐Con (Figure 2d,e). These results suggest that the engineered strain possesses the dual capability of engaging mucosal remnants and directly anchoring to exposed ECM proteins at sites of barrier disruption.

Most strikingly, in the Caco‐2 cell model representing direct host‐microbe interactions, plate colony counts revealed that EcN‐EB2 demonstrated a highly significant 11.87‐fold enhancement in attachment (Figure 2f). Inverted fluorescence microscopy further revealed that EcN‐EB2 formed dense aggregates across the Caco‐2 cell surfaces, whereas the control group exhibited only sporadic, non‐specific attachment (Figure 2g). Quantitative analysis confirmed that the EcN‐EB2 also exhibited significantly higher fluorescence intensity at the single‐cell level compared to the EcN‐Con (Figure S3). Collectively, these data provide compelling evidence that surface display of tSpaC successfully endows EcN with the capacity for precision targeting and localized retention within the inflamed intestinal microenvironment.

To further evaluate the in vivo performance of our adhesion‐enhanced platform, we investigated the intestinal retention and persistence of EcN‐EB2 in the gastrointestinal tract of healthy C57BL/6 mice. Mice were administered daily oral doses of either EcN‐Con or EcN‐EB2 (1 × 10^9^ CFU) for five consecutive days, with L‐(+)‐arabinose supplemented in the drinking water to ensure sustained induction of tSpaC expression. To visualize the spatial distribution of the engineered strains, an in vivo imaging system (IVIS) was conducted 24 h post‐final administration, revealing detectable fluorescence signals within the abdominal regions of both groups (Figure 2h). Although EcN‐EB2 showed a marginal increase in mean fluorescence intensity compared to EcN‐Con, the difference did not reach statistical significance (Figure 2i). To more precisely assess the colonic abundance and intestinal retention kinetics, we quantified viable bacteria recovered from faecal samples collected daily during the continuous 5‐day oral dosing period (Days 0–4) and throughout the 96 h post‐withdrawal washout phase (Days 5–8) (Figure S4). Both strains exhibited comparable accumulation levels during the treatment phase (Days 0–4). Following cessation of dosing, both groups showed a rapid decline in fecal bacterial loads. While EcN‐Con became undetectable by 72 h post‐administration (Day 7), EcN‐EB2 remained detectable for an additional 24 h, clearing completely by 96 h (Day 8). This modest enhancement in residence time, prolonging retention by only 24 h, likely stems from the scarcity of specific tSpaC‐binding targets such as exposed ECM in the intact healthy epithelium, coupled with potent colonization resistance exerted by the native microbiota. Importantly, the transient persistence of EcN‐EB2 in the healthy gut underscores its safety profile, as it ensures rapid elimination of the engineered strain in the absence of pathological inflammation.

To further evaluate the bio‐interfacial anchoring of the engineered probiotics on native tissues and to optimize the intervention dosage, we conducted an ex vivo colonic adhesion assay under both healthy and dextran sulphate sodium (DSS)‐induced inflammatory conditions (Figure S5). Quantitative fluorescence imaging confirmed that the adhesion‐enhanced strain EcN‐EB2 exhibited a significantly higher retention capacity on both healthy and inflamed colonic mucosa compared to the control strain EcN‐Con at a uniform dose of 1 × 10^9^ CFU (Figure S5a,c). Subsequent dose‐dependent evaluations ranging from 1 × 10^5^ to 1 × 10^10^ CFU were performed to investigate the binding kinetics. In the healthy model, bacterial adhesion reached a physical saturation plateau early at 1 × 10^6^ CFU, with no significant differences observed between the 1 × 10^6^ CFU group and any of the higher concentration groups (Figure S5b). In stark contrast, the DSS‐induced colitis model exhibited a higher binding capacity. In the inflamed colonic tissue, lower doses of 1 × 10^5^ and 1 × 10^6^ CFU failed to reach binding saturation. The physical saturation plateau was established at the 1 × 10^7^ CFU threshold, as no significant differences were observed between the 1 × 10^7^ CFU group and higher concentration groups (Figure S5d). Collectively, these distinct binding kinetics provide direct evidence for the inflammation‐targeted enrichment capability of the tSpaC‐displaying platform and establish the fundamental saturation limits necessary for guiding subsequent in vivo dosing regimens.

### 2.3 Systemic Biosafety Profiling of The Adhesion‐Enhanced EcN Platform

Although LGG is conventionally regarded as a safe probiotic, recent evidence has indicated that its SpaC pilin may trigger intestinal epithelial injury in zebrafish models via induction of cell pyroptosis and microbial dysbiosis [29]. These findings, together with the potential impact of surface‐display modifications on bacterial translocation, warranted a comprehensive safety assessment of our tSpaC‐expressing strain, EcN‐EB2. To assess its systemic biocompatibility, healthy C57BL/6 mice received daily oral gavage of EcN‐EB2 (1 × 10^9^ CFU) or PBS for seven consecutive days (Days 0–6), with L‐(+)‐arabinose supplemented in the drinking water to maintain continuous inducible expression of tSpaC. Throughout the treatment period, no significant fluctuations or differences in body weight were observed between the two groups (Figure S6). Upon sacrifice on day 7, routine blood test and biochemical indicator analysis revealed that all parameters remained within normal physiological ranges, with no discernible abnormalities detected in the EcN‐EB2‐treated group (Figure S7). Furthermore, the colon and major organs, including heart, liver, spleen, lungs, and kidneys, were histologically normal, with no evidence of tissue damage or inflammatory infiltration (Figure S8). To specifically address concerns regarding extra‐intestinal translocation, homogenates from systemic organs were plated on LB agar; notably, no viable EcN‐EB2 colonies were recovered from any of these non‐target tissues. Collectively, these results confirm that the adhesion‐enhanced EcN‐EB2 platform possesses excellent systemic biocompatibility, supporting its suitability for subsequent therapeutic applications.

### 2.4 Calprotectin‐Responsive Genetic Circuit for Autonomous ROS Scavenging and TFF3 Secretion

**FIGURE 3:**
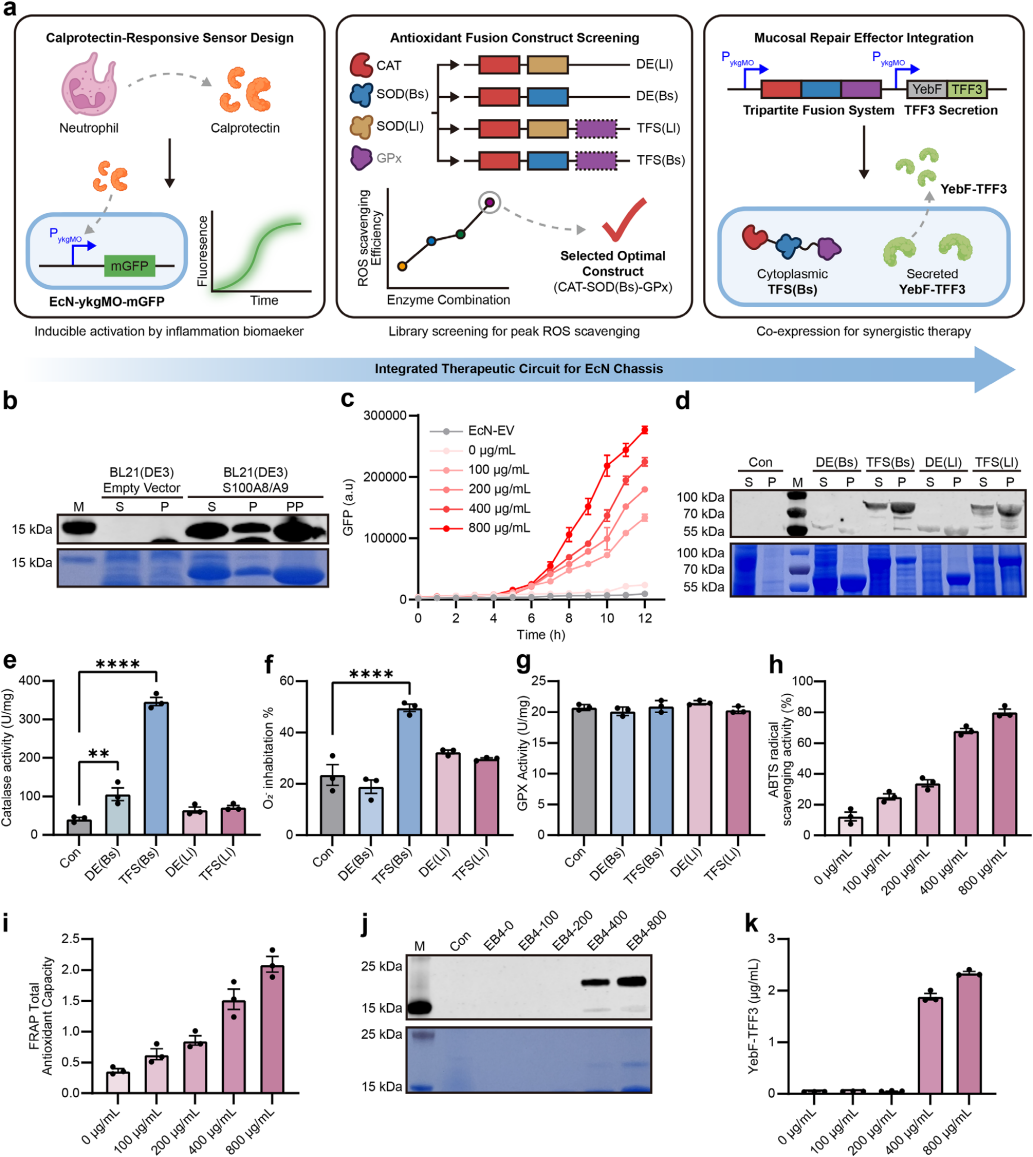
Engineering and characterization of the calprotectin‐responsive therapeutic circuit. (a) Hierarchical design of the autonomous therapeutic platform. The development process includes the design of an inflammation‐responsive sensor driven by the calprotectin‐sensitive *ykgMO* promoter. This stage is followed by screening of antioxidant fusion constructs to identify the CAT‐SOD(Bs)‐GPx tripartite fusion system, designated TFS(Bs), as the optimal candidate for peak ROS‐scavenging capacity. The final stage involves the genetic integration of the TFS(Bs)‐based antioxidant module and the YebF‐TFF3 secretion module to establish a synergistic therapeutic response within the EcN chassis. (b) Characterization of recombinant human calprotectin (S100A8/S100A9) expressed in *E. coli* BL21(DE3) via SDS‐PAGE and Western blot. (c) Dose‐dependent activation of the *ykgMO* promoter by recombinant calprotectin (0–800 µg/mL), quantified via mNeonGreen (mGFP) reporter fluorescence. (d–g) Screening of the CAT‐SOD dual‐enzyme (DE) and CAT‐SOD‐GPx tripartite fusion system (TFS) constructs. Expression levels were analyzed by SDS‐PAGE and Western blot (d), followed by individual activity assays for Catalase (e), SOD (f), and GPx (g). (h,i) Total antioxidant capacity (T‐AOC) of the optimized TFS system induced by indicated calprotectin concentrations, measured by ABTS (h) and FRAP (i) assays. (j, k) Secretion of YebF‐TFF3 in EcN‐EB4. Immunoblots of concentrated culture supernatants (j) and ELISA quantification of secreted hTFF3 (k) under calprotectin induction. Data are presented as mean ± SEM (*n* = 6 independent biological replicates for c; *n* = 3 independent biological replicates for e–i, k). Statistical significance was determined using a one‐way ANOVA followed by Tukey's post‐hoc test (^****^ *p* < 0.0001; ^**^ *p* < 0.01).

Leveraging the engineered probiotic EcN‐EB2 platform, we sought to transform this programmable, adhesion‐enhanced probiotic platform into an autonomous “sense‐and‐respond” LBP for the inflammation‐responsive management of IBD (Figure 3a). To circumvent the metabolic burden and potential off‐target effects of constitutive expression, we integrated a calprotectin‐responsive circuit driven by the *ykgMO* promoter [19]. The closed‐loop system regulates a dual‐pronged therapeutic response consisting of a CAT‐SOD‐GPx TFS‐based antioxidant module and a YebF‐mediated TFF3 secretion module. This integrated architecture was designed to initiate therapeutic intervention exclusively upon detection of inflammatory cues, thereby facilitating localized restitution of the compromised intestinal barrier.

To assess the responsiveness of the engineered *ykgMO* promoter, we first performed heterologous expression and purification of recombinant calprotectin in *E. coli* BL21(DE3). Calprotectin is a heterodimeric complex composed of S100A8 (GenBank: CR407674.1) and S100A9 (GenBank: CR542224.1), which possesses the intrinsic ability to undergo spontaneous assembly [30, 31]. In this study, we implemented a single‐strain strategy for synthesis of the intact recombinant heterodimer. Briefly, codon‐optimized S100A8 and S100A9 genes were subcloned into the pRSFDuet‐1 dual‐expression vector, thereby facilitating their spontaneous intracellular association during co‐expression. A 6 × His tag was fused to the N‐terminus of S100A8 to enable specific detection and affinity purification. SDS‐PAGE and Western blot analyses of cell lysates and Ni‐NTA purified fractions confirmed the successful production of recombinant calprotectin (Figure 3b). Under the heat‐denaturing conditions employed for analysis, the heterodimer predominantly dissociated into its constituent monomers, yielding characteristic bands for both subunits in both the lysate supernatants and purified protein fractions. Notably, the presence of trace undissociated complexes in the purified protein lane further corroborated successful molecular assembly of the recombinant heterodimer (Figure S9).

To quantitatively evaluate the sensing performance of the *ykgMO* promoter, we developed a reporter strain, EcN‐ykgMO‐mGFP, by placing the mGFP gene under its transcriptional control. This architecture enabled measurement of promoter activity as a function of fluorescence intensity. Upon exposure to various concentrations of recombinant calprotectin (100–800 µg/mL), the reporter strain exhibited a robust and dose‐dependent increase in fluorescence (Figure 3c). Specifically, at 12 h post‐induction, treatment with 100, 200, 400, and 800 µg/mL calprotectin resulted in 5.57‐, 7.50‐, 9.37‐, and 11.54‐fold increases in fluorescence intensity, respectively, relative to the untreated control group. These findings validate the *ykgMO* promoter as an effective and sensitive biosensing element for calprotectin within the EcN chassis, providing a reliable foundation for the autonomous activation of the downstream therapeutic modules.

To construct the antioxidant module, we developed two classes of fusion constructs: a CAT‐SOD dual‐enzyme fusion (DE) and an expanded CAT‐SOD‐GPx tripartite fusion system (TFS). The DE systems were constructed by fusing CAT from *Lactobacillus plantarum* ATCC 14431 with SOD derived from either *Bacillus subtilis* 168 (Bs) or *Lactococcus lactis* NZ9000 (Ll) via rigid linkers. A putative GPx from *Lactobacillus casei* ATCC 393 was subsequently integrated into the corresponding DE scaffolds via flexible linkers to generate TFS(Bs) and TFS(Ll), with a Flag tag fused to the CAT N‐terminus for immunodetection [32].

To evaluate the functional integration of these fusion constructs, we systematically characterized their expression profiles and catalytic activities. Assessment by SDS‐PAGE and immunoblotting revealed that both DE(Bs) and DE(Ll) constructs exhibited extremely poor soluble expression, predominantly partitioning into inclusion bodies with only marginal signals detectable in the soluble fraction (Figure 3d). This observation suggested that the direct fusion of CAT and SOD incurred significant proteostatic stress, leading to pervasive misfolding. Intriguingly, incorporation of the putative GPx into the DE framework to generate the TFS constructs dramatically mitigated this folding bottleneck. Both TFS(Bs) and TFS(Ll) exhibited markedly enhanced soluble expression alongside a reciprocal reduction in aggregate formation (Figure 3d). Although enzymatic profiling confirmed that the integrated GPx lacked discernible catalytic activity, suggesting that the detected background signal likely originated from endogenous EcN enzymes (Figure 3g), its incorporation was associated with markedly improved soluble expression and reduced aggregation, suggesting a potential structural‐support role within the fusion scaffold. Specifically, GPx may function as a chaperone‐like fusion partner that facilitates the folding and structural maturation of the CAT‐SOD complex. This structural optimization manifested as a profound enhancement in catalytic capacity: while CAT activity remained modest in the DE formats, TFS(Bs) system achieved a 3.28‐fold increase in CAT activity relative to its DE(Bs) predecessor (Figure 3e). Similarly, despite the lower baseline activity of SOD(Bs) compared to SOD(Ll) in the DE configuration, the improved folding of TFS(Bs) elicited a 2.62‐fold elevation in SOD activity, representing the highest superoxide‐scavenging capacity among all candidate constructs (Figure 3f). Consequently, EcN‐TFS(Bs) was identified as the optimal therapeutic module for subsequent antioxidant interventions.

Next, the TFS(Bs)‐based antioxidant module was genetically coupled downstream of the *ykgMO* promoter within the pET‐tSpaC‐mGFP backbone, yielding the recombinant strain EcN‐EB3. Induction with calprotectin (0–800 µg/mL) triggered a concentration‐dependent increase in total antioxidant activity as measured by ABTS and FRAP assays (Figure 3h,i). These results demonstrate that the TFS‐based antioxidant module can be successfully and responsively activated by the inflammatory biomarker calprotectin to exert its biochemical functions. In summary, these results establish that EcN‐EB3 implements an autonomous antioxidant response precisely gated by inflammatory biomarkers.

To further augment the capacity of the engineered platform for regenerating the damaged intestinal barrier, we developed a mucosal repair module utilizing YebF‐mediated secretion of TFF3 [9, 33, 34]. In this design, the N‐terminus of TFF3 was fused to the *E. coli* secretory carrier protein YebF to facilitate extracellular translocation, while a 6 × His tag was incorporated at the C‐terminus for detection and purification. This secretory assembly was placed under the transcriptional control of the *ykgMO* promoter and integrated into the EcN‐EB3 platform to generate the recombinant strain EcN‐EB4. To evaluate the secretion profile across a range of inflammatory stimuli, EcN‐EB4 was cultured with recombinant calprotectin concentrations ranging from 0 to 800 µg/mL. The resulting culture supernatants were harvested and concentrated via ultrafiltration for subsequent analysis. SDS‐PAGE and Western blot assays confirmed expression of the YebF‐TFF3‐His fusion protein, with distinct bands exclusively observed at calprotectin concentrations of 400 and 800 µg/mL (Figure 3j). This threshold‐dependent detection was further corroborated by ELISA quantification of the secreted TFF3 (Figure 3k). These observations likely reflect either low‐level expression below the detection limit at lower inducer concentrations or potential extracellular degradation of the fusion protein. Consequently, these findings establish that EcN‐EB4 can successfully mediate TFF3 secretion within the active inflammatory environments characteristic of severe disease states.

### 2.5 Improved Gastrointestinal Survival Through Enteric Microencapsulation

**FIGURE 4:**
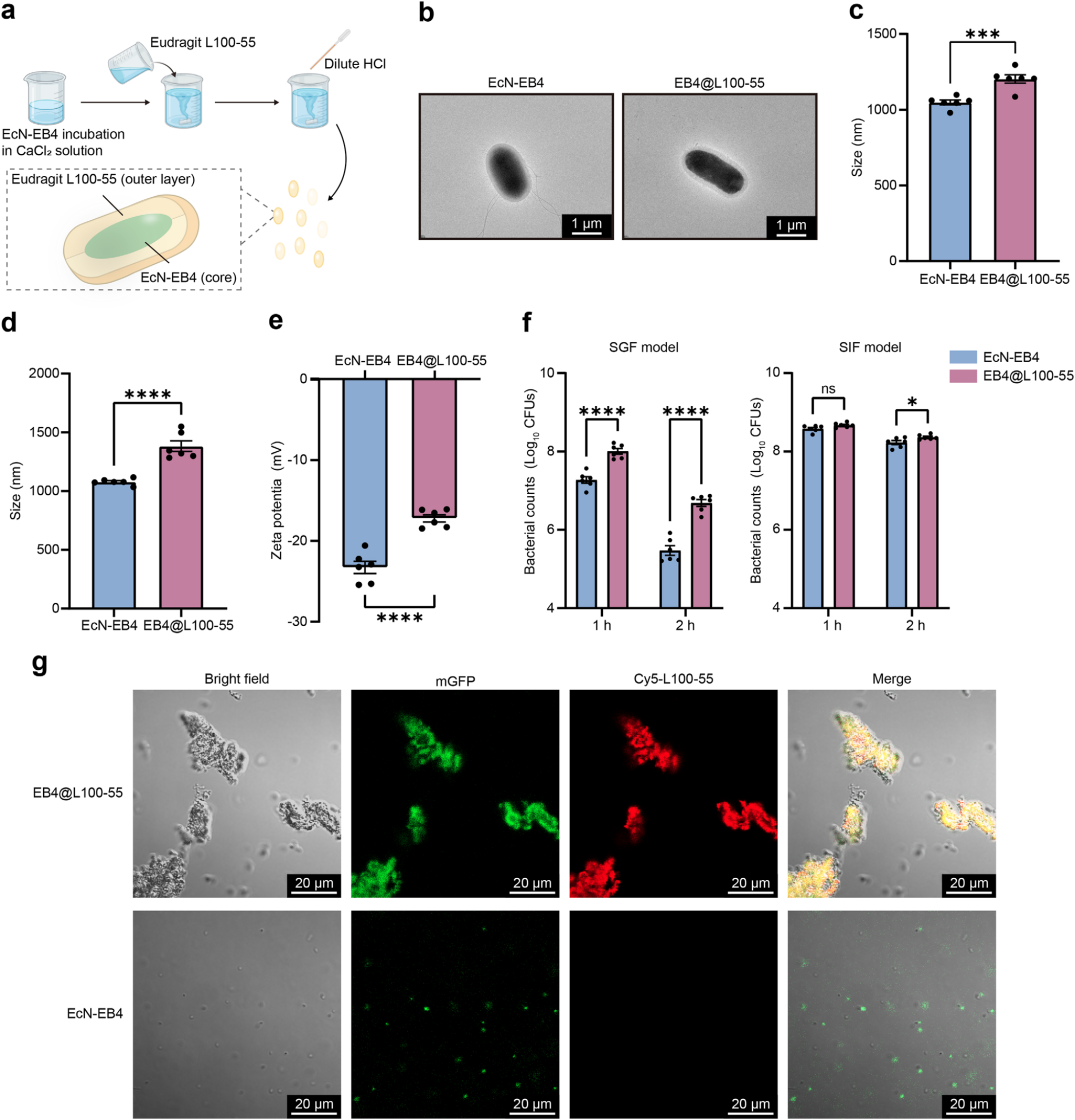
Physical characterization and gastrointestinal stability of microencapsulated EcN‐EB4. (a) Schematic illustration of the microencapsulation process of engineered EcN‐EB4 strains using Eudragit L100‐55 (L100‐55). (b) Representative transmission electron microscopy (TEM) images of EcN‐EB4 and encapsulated EcN‐EB4 (EB4@L100‐55). Scale bar, 1 µm. (c) Quantitative analysis of bacterial width based on TEM images to evaluate the thickness of the L100‐55 coating (*n* = 6). (d,e) Hydrodynamic diameter (d) and zeta potential (e) of EcN‐EB4 and EB4@L100‐55 measured by dynamic light scattering (DLS). (f) Survival and stability of encapsulated bacteria in simulated gastric fluid (SGF) and simulated intestinal fluid (SIF) models at indicated time points. (g) Confocal laser scanning microscopy (CLSM) images of EB4@L100‐55. The L100‐55 coating is labeled with Cy5 (red), and EcN‐EB4 expresses mGFP (green). Scale bar, 20 µm. Data are presented as mean ± SEM (*n* = 6 independent biological replicates for all quantitative data and representative microscopy images). Statistical significance was determined using a two‐tailed Student's t‐test (c–e) or multiple t‐tests (f) (^****^ *p* < 0.0001; ^***^ *p* < 0.001; ^*^ *p* < 0.05; ns, not significant).

To protect EcN‐EB4 from gastric degradation, we encapsulated the strain within a pH‐responsive Eudragit L100‐55 shell based on established enteric coating protocols [35, 36]. The hierarchical assembly process of the resulting EB4@L100‐55 is schematically illustrated in Figure 4a. Morphological analysis via transmission electron microscopy (TEM) revealed that uncoated EcN‐EB4 retained its characteristic fimbriae and flagella, whereas the encapsulated variants were fully enveloped by a distinct nanocoating that transformed the bacterial surface into a wrinkled shell architecture (Figure 4b). Quantitative cross‐sectional analysis confirmed that the bacterial thickness significantly expanded from 1039.01 ± 59.02 to 1191.01 ± 106.01 nm following the interfacial coating process (Figure 4c). Consistently, dynamic light scattering (DLS) measurements showed a marked increase in average hydrodynamic diameter from 1075.5 ± 41.5 to 1416.5 ± 132.5 nm (Figure 4d). This physical integration was further accompanied by a shift in Zeta potential from −23.01 ± 2.42 to −17.30 ± 1.12 mV (Figure 4e), reflecting the successful interfacial assembly of the anionic polymer. To verify the uniformity and distribution of the coating, we performed laser scanning confocal microscopy (LSCM) using Cy5‐labeled L100‐55, where the colocalization of the intracellular mGFP signal with the extracellular Cy5 red fluorescence confirmed that engineered probiotics were shielded by the enteric shell (Figure 4g).

The functional protective capacity and pH‐triggered release profile of the platform were evaluated through in vitro digestion assays in simulated gastric fluid (SGF, pH 2.0) and simulated intestinal fluid (SIF, pH 7.0) (Figure 4f). In the SGF model, the L100‐55 coating exerted profound protective effects at both 1 and 2 h intervals. Notably, after 2 h of acidic exposure, the viable count of EB4@L100‐55 was one order of magnitude higher than that of the uncoated group (10^6^ vs. 10^5^ CFU), underscoring the efficiency of the polymer barrier in preserving bacterial integrity against gastric insults. Conversely, in the SIF environment, the encapsulated bacteria exhibited proliferation rates and viable counts nearly identical to those of the control group. These findings demonstrate that the Eudragit L100‐55 shell undergoes rapid and complete disintegration under intestinal pH conditions without imposing long‐term inhibitory effects on bacterial fitness or growth potential.

To evaluate the stringency of the inducible display system and the protective efficacy of enteric microencapsulation during gastrointestinal transit, we monitored surface tSpaC‐positive rates via flow cytometry following immunofluorescent labeling (Figure S10). The uninduced EcN‐EB4(L‐ara‐) exhibited negligible fluorescence comparable to the control strain, confirming the stringent regulation of the arabinose promoter with almost no basal leaky expression. Upon induction, the unencapsulated strains (EcN‐EB2 and EcN‐EB4) achieved robust initial display efficiencies of 80.99% and 80.43%, respectively; however, their exposed adhesins were severely compromised during sequential SGF and SIF digestion. Conversely, the EB4@L100‐55 formulation demonstrated robust pH‐responsive shielding and release kinetics. The physical barrier of the polymer shell initially precluded fluorescent antibody binding, yielding a low baseline signal. Following strong structural preservation throughout the acidic SGF phase, the enteric capsule rapidly disintegrated in the neutral SIF environment. This triggered the release of intact engineered bacteria, facilitating a significant recovery of the tSpaC‐positive rate to 74.98%. This molecular‐level tracking robustly validates that the microencapsulation strategy effectively preserves the adhesive functionality of the probiotics, facilitating their enhanced intestinal retention.

To verify the adhesion and intestinal retention efficiency of the engineered bacteria in an in vivo model, we performed ex vivo fluorescence imaging of the gastrointestinal tract 4 h after a single oral administration in mice with DSS‐induced colitis (Figures S11 and S12). The imaging results revealed that the PBS and EcN‐Con groups exhibited negligible fluorescence. In contrast, the EcN‐EB4 and EB4@L100‐55 groups displayed significant fluorescence accumulation in the distal colon, with the signal in the EB4@L100‐55 group being notably more intense (Figure S11). Quantitative analysis further confirmed that the surface displayed tSpaC markedly enhanced intestinal retention, while the superior performance of the EB4@L100‐55 group highlights the protective role of the Eudragit coating during gastrointestinal transit (Figure S12). These findings demonstrate that the engineered probiotics can successfully achieve adhesion‐enhanced intestinal retention within the inflammatory intestinal environment.

### 2.6 Therapeutic Efficacy of Programmable EcN in Alleviating DSS‐Induced Murine Colitis

**FIGURE 5:**
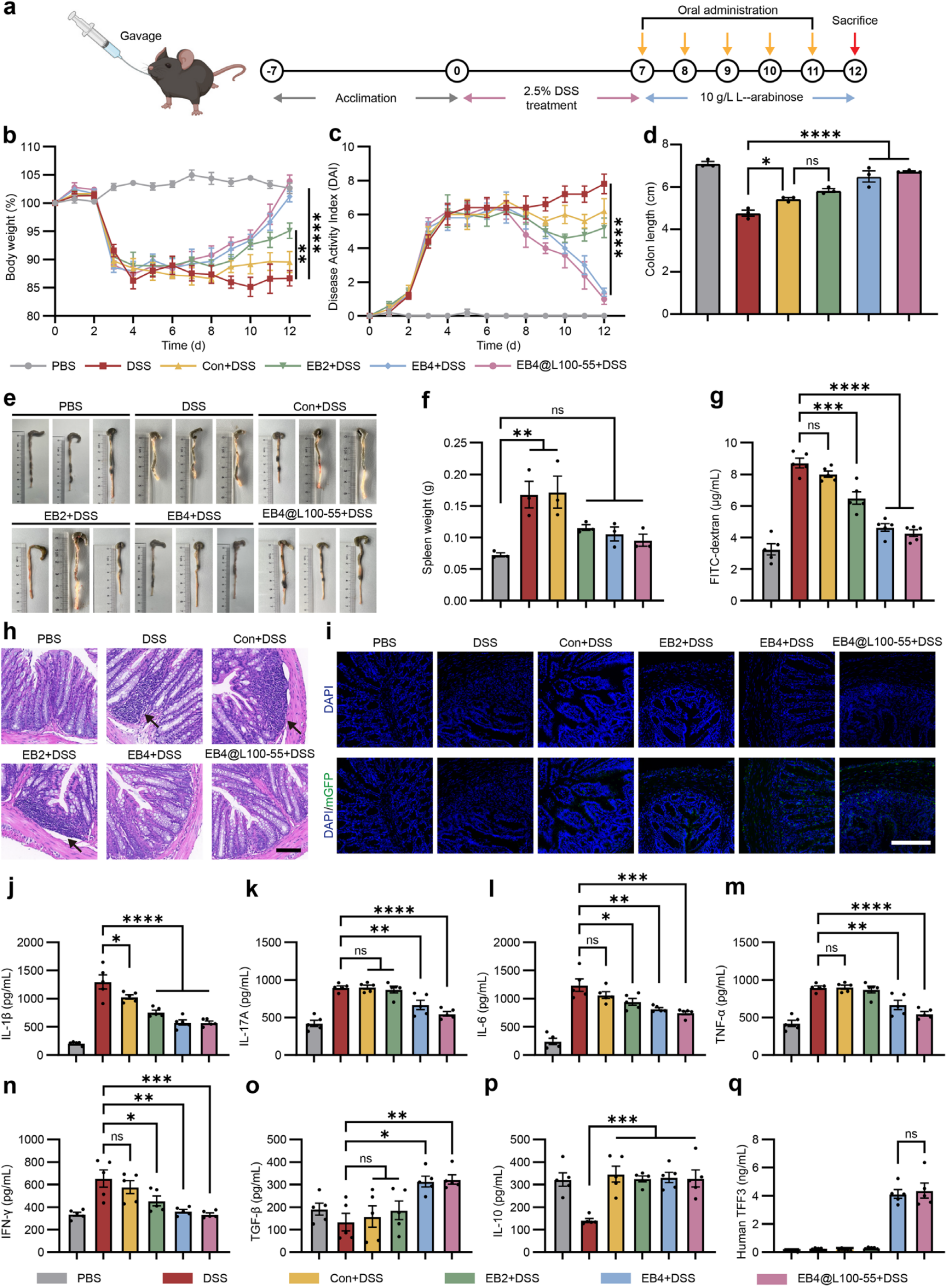
Therapeutic efficacy of microencapsulated EB4 in the delayed treatment colitis model. (a) Schematic representation of the DSS‐induced colitis induction and the delayed treatment protocol. (b, c) Body weight change curves (b) and disease activity index (DAI) scores (c) recorded over the 12‐day experimental period. (d, e) Statistical analysis of colon length (d) and representative macroscopic images of colons (e) harvested on day 12. (f) Spleen weights from mice in the indicated experimental groups. (g) Intestinal permeability assessed by serum FITC‐dextran levels following oral administration. (h) Representative H&E‐stained images of colon sections showing histopathological changes. Black arrows indicate inflammatory infiltration. Scale bars, 100 µm. (i) Representative fluorescence images showing the distribution of mGFP‐labeled engineered bacteria in colon tissues. Scale bar, 200 µm. (j–p) ELISA quantification of pro‐inflammatory and anti‐inflammatory cytokines in colon tissues. (q) ELISA quantification of secreted human TFF3 in colon tissues. Data are presented as mean ± SEM (*n* = 3 biologically independent animals for d and f; *n* = 5 biologically independent animals for b, c, g, and j to q). For all representative macroscopic and microscopic images (e, h, and i), results were confirmed in at least three independent animals per group. Statistical significance was determined via one‐way ANOVA followed by Tukey's post‐hoc test (^****^ *p* < 0.0001; ^***^ *p* < 0.001; ^**^ *p* < 0.01; ^*^ *p* < 0.05; ns, not significant).

To evaluate the therapeutic efficacy of our engineered probiotics in a murine IBD model, we established a challenging delayed‐treatment model using DSS‐induced acute colitis in C57BL/6 mice (Figure 5a). The control group (PBS) received regular drinking water, while the DSS‐treated group and engineered probiotic‐treated groups were induced with 2.5% DSS in their drinking water for 7 consecutive days. During the subsequent therapeutic phase (Day 7–11), the engineered probiotic‐treated groups were stratified into four cohorts. For clarity and simplicity in all following in vivo sections and corresponding figures, the engineered strains EcN‐Con, EcN‐EB2, and EcN‐EB4 are hereafter abbreviated as Con, EB2, and EB4, respectively. Thus, the four specific intervention cohorts were designated as Con+DSS, EB2+DSS, EB4+DSS, and the Eudragit L100‐55 encapsulated formulation as EB4@L100‐55+DSS. These mice were administered 1 × 10^9^ CFU of their respective probiotics via daily oral gavage, while the PBS and DSS groups received gavage with PBS buffer to ensure a consistent stimulus. Throughout the therapeutic phase, the drinking water was supplemented with L‐(+)‐arabinose to maintain the sustained induction of surface‐displayed tSpaC.

The successful establishment of the inflammatory model was evidenced by significant body weight loss and a marked elevation in the disease activity index (DAI) following continuous DSS treatment. As shown in Figure 5b, during the therapeutic phase, the Con group and the adhesion‐enhanced EB2 group showed extremely limited signs of recovery. In contrast, the integrated platforms (EB4 and EB4@L100‐55) combining ROS‐scavenging and TFF3‐secreting modules significantly arrested the weight loss trend and reduced DAI scores, demonstrating superior clinical intervention potential. Mice were euthanized on Day 12 to assess colon length, spleen weight, and intestinal permeability (Figure 5d–g). DSS‐induced inflammation led to severe colon shortening and significant splenomegaly, reflecting intense localized pathology and systemic inflammation (Figure 5d–f and Figure S13). Notably, colon lengths were effectively maintained in the EB4 and EB4@L100‐55 groups, with spleen weights recovering to levels comparable to healthy controls. Furthermore, analysis of serum FITC‐dextran (FD4) fluorescence intensity showed that while the EB2 group provided a modest improvement in barrier integrity, the EB4‐series strains achieved the most significant reduction in permeability, effectively repairing the damaged epithelial structure (Figure 5g). Histopathological evaluation further confirmed the extensive epithelial barrier destruction and inflammatory cell infiltration in the DSS group. Treatment with EB4 and EB4@L100‐55 preserved the integrity of the epithelial barrier and substantially reduced cell infiltration, thereby improving the overall pathological architecture of the colonic tissue (Figure 5h and Figure S14). Given that intestinal persistence is a prerequisite for therapeutic efficacy, we assessed the actual impact of the adhesion‐enhancement strategy on the intestinal retention of engineered probiotics. Fluorescence scanning of colonic sections revealed prominent signals in the EB2 and its derivative groups (Figure 5i). To continuously monitor bacterial transit and accumulation, fecal samples were collected daily prior to each gavage (Days 7–11), with a final sample collected at the time of sacrifice on Day 12 (24 h post‐final administration). Plate counts of these samples confirmed the enhanced intestinal retention efficiency of these tSpaC‐displaying strains (Figure S15). These results demonstrate that surface‐display‐mediated adhesion enhancement successfully promotes probiotic enrichment at the inflamed mucosa, providing a stable bioprocessing platform for subsequent ROS scavenging and tissue repair functions.

We then investigated the mechanisms underlying ROS neutralization and barrier restoration. Fluorescence probe scanning and MPO activity assays indicated that the EB4‐series strains accurately cleared the abnormally elevated ROS in the inflammatory environment, thereby reversing oxidative damage (Figures S16 and S17). This improvement in the local microenvironment facilitated barrier restoration, as evidenced by immunofluorescence staining, which showed that the EB4@L100‐55 group exhibited a continuous distribution pattern of the tight junction proteins Claudin, Occludin, and ZO‐1 along the intercellular junctions. This restorative pattern suggests that the engineered platform effectively reinforced cell‐cell junctions and achieved the functional repair of the colonic epithelial seal (Figure S18). Finally, the expression levels of inflammation‐related cytokines in colonic tissue were analysed by ELISA (Figure 5j–q). DSS treatment triggered a typical pro‐inflammatory cascade, leading to a drastic rise in IL‐1β, TNF‐α, and IL‐6. Notably, while all engineered strains exerted some degree of regulation, the pronounced downregulation of IL‐17A and TNF‐α, alongside a significant recovery of the anti‐inflammatory cytokine TGF‐β, was primarily observed in the EB4 and EB4@L100‐55 groups. These results suggest that engineered probiotics integrating ROS‐scavenging and TFF3‐secretion modules can precisely respond to inflammatory cues and achieve potent remission of colitis through the combined action of mucosal anchoring, antioxidant activity, and epithelial repair.

### 2.7 Gut Microbiota Modulation in The Therapeutic Model

**FIGURE 6:**
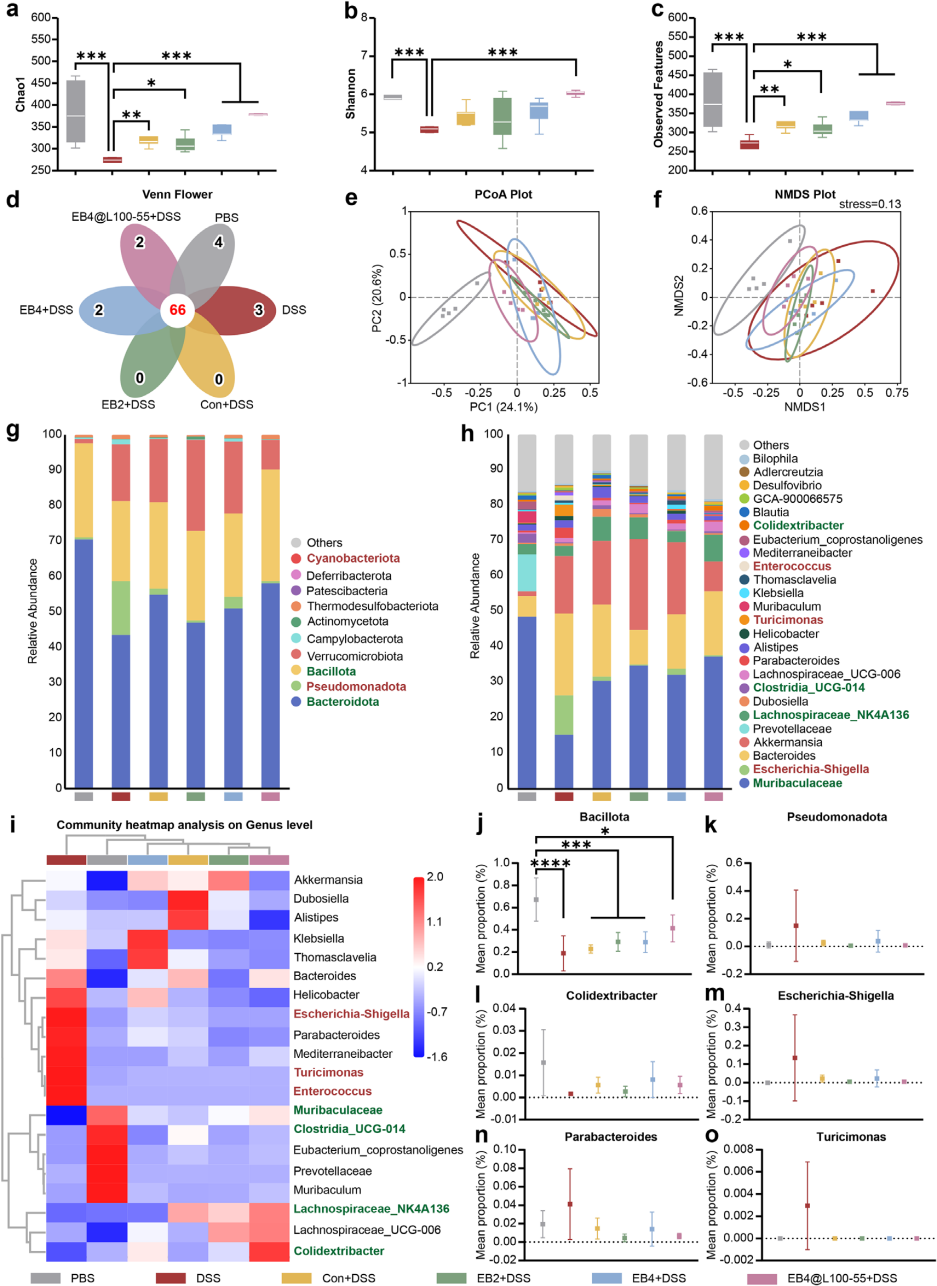
16S rRNA sequencing analysis of gut microbiota composition in the delayed treatment model. (a–c) Microbial α‐diversity assessed via Chao1 (a), Shannon (b), and observed features (c) indices across the experimental groups. (d) Venn flower plot illustrating the overlap of operational taxonomic units (OTUs) among different groups. (e,f) Microbial β‐diversity visualized by Principal Coordinate Analysis (PCoA) based on Bray‐Curtis distance (e) and Non‐metric Multidimensional Scaling (NMDS) plot (f; stress = 0.13). Ellipses denote 95% confidence intervals. (g, h) Relative abundance of gut microbiota at the phylum level (g, top 10) and genus level (h, top 25). (i) Community heatmap analysis displaying the relative abundance of the top 20 genera across different groups. (j,k) Mean proportions of the phyla *Bacillota* (j) and *Pseudomonadota* (k). (l–o) Mean proportions of selected genus‐level taxa, including *Colidextribacter* (l), *Escherichia‐Shigella* (m), *Parabacteroides* (n), and *Turicimonas* (o). Data are presented as mean ± SD from *n* = 5 biologically independent animals per group. Statistical significance was determined via one‐way ANOVA followed by Tukey's post‐hoc test (^****^ *p* < 0.0001; ^***^ *p* < 0.001; ^**^ *p* < 0.01; ^*^ *p* < 0.05; ns, not significant). The names of key biologically relevant taxa are highlighted in bold, with anti‐inflammatory and beneficial taxa color coded in green, and pro‐inflammatory or pathobiont taxa marked in red.

To evaluate the structural shifts in the gut microbiota following probiotic intervention, we performed 16S rRNA gene sequencing on colonic contents. After OTU clustering, we first assessed the α diversity to examine microbial richness and evenness across the different treatment groups. Results from Chao1, Shannon, and Observed features indices, alongside the Simpson index, collectively demonstrated a significant reduction in microbial richness in the DSS‐treated group compared to the healthy PBS groups (Figure 6a–c and Figure S19). Notably, all engineered probiotic interventions facilitated the restoration of microbial diversity. Encouragingly, the integrated EB4 and EB4@L100‐55 platforms, equipped with inflammation‐responsive therapeutic modules, exhibited superior optimization of microbial diversity compared to the adhesion‐enhanced chassis EB2. A Venn diagram identified 66 shared OTUs across all groups, with only a limited number of unique OTUs in the DSS (3) and EB4@L100‐55 (2) cohorts, suggesting that the primary impact of treatment lies in the redistribution of existing microbial abundance rather than a total shift in OTU composition (Figure 6d). Structural divergence was further characterized via β diversity analysis. PCoA, NMDS, and PCA plots revealed distinct separation between the PBS and DSS groups, confirming that DSS treatment caused a profound shift in β diversity (Figure 6e,f and Figure S20). Specifically, PCoA and NMDS plots revealed that the EB4@L100‐55 group showed a distinct migration from the diseased state (DSS group) toward the healthy PBS cluster. This result demonstrates the potent regulatory capacity of our multi‐modal strategy in rectifying microbial imbalances. Further analysis of the microbial composition at phylum level revealed that DSS treatment reduced the dominance of *Bacteroidota* and *Bacillota* while promoting the expansion of *Pseudomonadota* and *Cyanobacteriota* (Figure 6g). Engineered probiotic interventions effectively reversed these trends by promoting the recovery of *Bacteroidota* and suppressing the overgrowth of inflammatory‐associated *Pseudomonadota*. At the genus level, DSS treatment induced a significant depletion of obligate anaerobes including *Muribaculaceae* and *Prevotellaceae* alongside an enrichment of pathobionts such as *Escherichia‐Shigella* and *Turicimonas* (Figure 6h). The EB4 and EB4@L100‐55 strains reversed these microbial alterations. Notably, EB2 and EB4‐series strains increased the abundance of *Colidextribacter*. Heatmap analysis of the top 20 genera corroborated these observations, revealing reduced enrichment of *Enterococcus* and *Escherichia‐Shigella* upon treatment. Although the probiotics did not fully restore the levels of *Muribaculaceae* or *Clostridia*_UCG‐014 to baseline, EB4@L100‐55 markedly enriched *Lachnospiraceae*_NK4A136 (Figure 6i), a family critical for dietary fibre degradation and butyrate production to support epithelial health. Subsequently, individual analysis of key taxa indicated a trend toward the restoration of *Bacillota* and *Colidextribacter* following engineered probiotic treatment, while *Pseudomonadota* and *Escherichia‐Shigella* showed a clear downward trend toward baseline levels (Figure 6j–o). Finally, ternary plot analysis further validated the differential enrichment of taxa. Compared to the DSS group, EB4 and EB4@L100‐55 effectively inhibited *Pseudomonadota* and *Escherichia‐Shigella* (Figure S21). These findings demonstrate that the integrated EB4@L100‐55 platform induces robust remission of colitis not only through direct immunomodulation but also by remodeling the colonic microenvironment and rebalancing the gut microbiota.

To further elucidate the ecological remodeling, quantitative comparative analyses were performed at the genus level (Figure S22). Specifically, compared to the DSS induced disease group, the relative abundance of *Lachnospiraceae*_NK4A136 demonstrated a statistically significant restoration following EcN‐EB4@L100‐55 intervention. Additionally, treatment with either EcN‐EB4 or EcN‐EB4@L100‐55 mediated a mean recovery in the abundance of *Colidextribacter* and concurrently constrained the expansion of *Escherichia‐Shigella*. Furthermore, *Turicimonas* was exclusively identified within the DSS challenged group and remained completely undetectable across all other experimental groups. Collectively, these specific taxonomic shifts provide evidence that the engineered platforms effectively drive the structural rehabilitation of the intestinal microbiota.

To functionally evaluate the metabolic shifts associated with the altered microbiota, fecal butanoic acid was specifically quantified via quantitative analysis (Figure S23). Consistent with the structural dysbiosis, DSS induction led to a severe depletion of fecal butyrate. However, intervention with the engineered probiotics effectively reversed this metabolic defect, driving a significant upward trend in butyrate levels. Notably, both EcN‐EB4 and EB4@L100‐55 exhibited a remarkable improvement against inflammation induced metabolite loss. The fully integrated EB4@L100‐55 platform achieved the most substantial metabolic recovery. Although its butyrate concentration remained marginally below the absolute healthy baseline, it was drastically elevated compared to the disease group and significantly outperformed the unmodified EcN‐Con strain. This outcome definitively demonstrates that the modular engineering modifications combined with microencapsulation profoundly enhance the capacity of the chassis strain to alleviate inflammation and rescue gut metabolism. Ultimately, the significant recovery of butanoic acid functionally corroborates the overall improvement in the intestinal microecological structure, suggesting that the engineered platform not only remodels the microbial composition but also reinstates the essential metabolic functions required for mucosal healing.

### 2.8 Prophylactic Efficacy of Programmable EcN Against DSS‐Induced Murine Colitis

**FIGURE 7:**
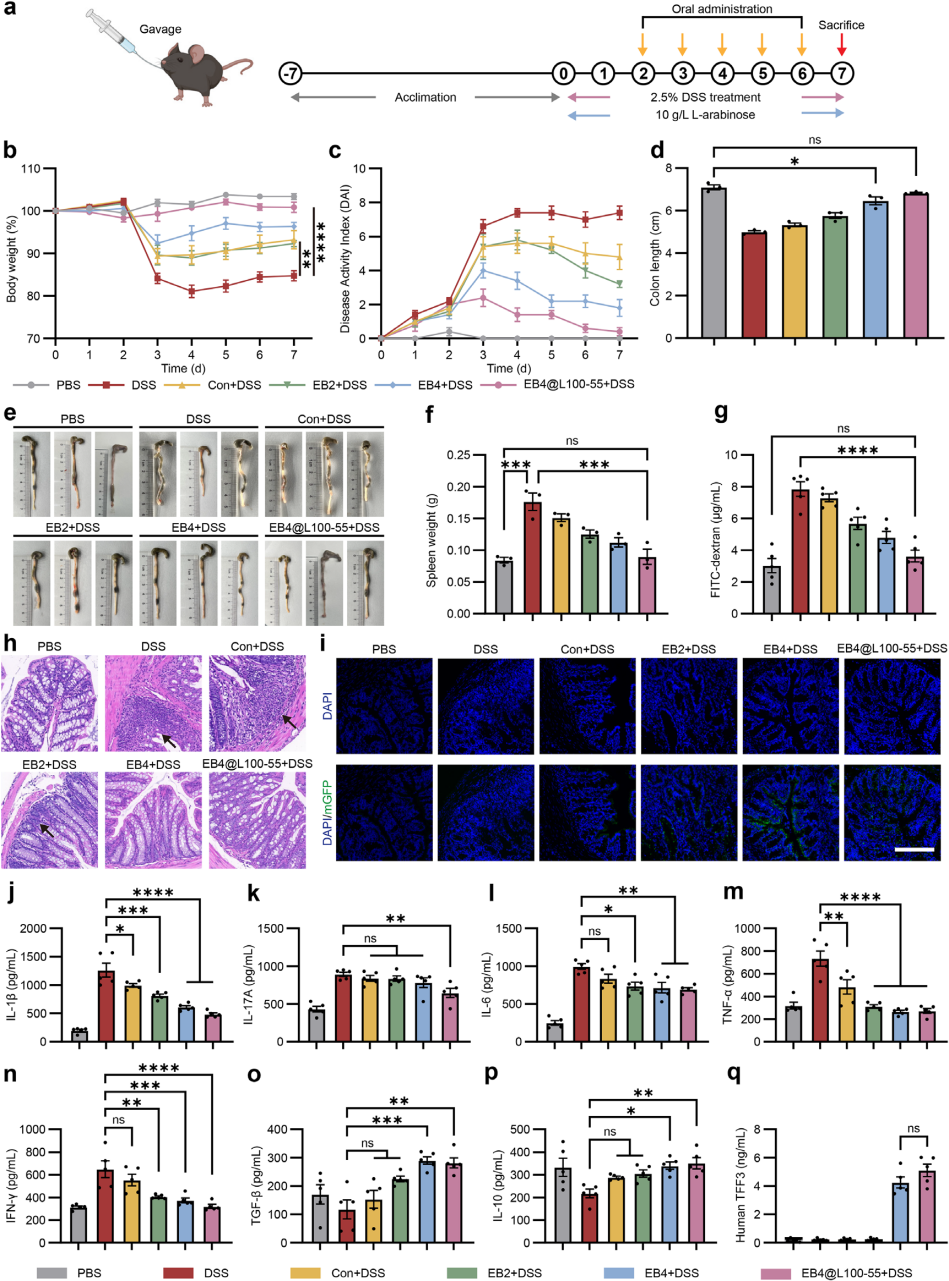
Therapeutic efficacy of microencapsulated EB4 in the prophylactic colitis model. (a) Schematic representation of the DSS‐induced colitis induction and the prophylactic treatment protocol. (b, c) Body weight change curves (b) and disease activity index (DAI) scores (c) recorded over the 7‐day experimental period. (d,e) Statistical analysis of colon length (d) and representative macroscopic images of colons (e) harvested on day 7. (f) Spleen weights from mice in the indicated experimental groups. (g) Intestinal permeability assessed by serum FITC‐dextran levels following oral administration. (h) Representative H&E‐stained images of colon sections showing histopathological changes. Black arrows indicate inflammatory infiltration. Scale bars, 100 µm. (i) Representative fluorescence images showing the distribution of mGFP‐labeled engineered bacteria in colon tissues. Scale bar, 200 µm. (j–p) ELISA quantification of pro‐inflammatory and anti‐inflammatory cytokines in colon tissues. (q) ELISA quantification of secreted human TFF3 in colon tissues. Data are presented as mean ± SEM (*n* = 3 biologically independent animals for d and f; *n* = 5 biologically independent animals for b, c, g, and j to q). For all representative macroscopic and microscopic images (e, h, and i), results were confirmed in at least three independent animals per group. Statistical significance was determined via one‐way ANOVA followed by Tukey's post‐hoc test (^****^ *p* < 0.0001; ^***^ *p* < 0.001; ^**^ *p* < 0.01; ^*^ *p* < 0.05; ns, not significant).

Given the robust efficacy of our engineered probiotics in treating established disease, we further explored their potential as a preventive intervention by establishing a prophylactic model during the early stages of IBD pathogenesis (Figure 7a). Acute colitis was induced in C57BL/6 mice by administering 2.5% DSS in the drinking water. In contrast to the delayed‐treatment model, mice in the intervention groups received daily oral gavage of probiotics starting from the second day of DSS treatment, with 10 g/L L‐(+)‐arabinose co‐administered in the drinking water to upregulate adhesion‐related functional protein expression. Throughout the experimental period, body weight and DAI scores were monitored. Encouragingly, the oral administration of engineered probiotics during the early disease course provided a profound protective effect against progression. As shown in Figure 7b, intervention with EB4@L100‐55 successfully stabilized the body weight of mice, effectively forestalling the downward trend observed in other DSS‐treated groups. The DAI scores exhibited a distinct gradient of improvement as the engineered probiotics were upgraded. By the final day of this model (Day 7), the DAI scores in the EB4@L100‐55 cohort had returned to levels with no significant difference compared to the healthy PBS controls (Figure 7c). These findings were further corroborated by assessments of colon length, spleen weight, and intestinal permeability. Specifically, the EB4@L100‐55 group effectively maintained physiological colon length and prevented splenomegaly, with intestinal permeability also returning to levels identical to the healthy PBS group (Figure 7d–g, Figure S24). Histopathological evaluation provided microscopic evidence of this preventive shield. While the Con and EB2 groups showed limited capacity to inhibit inflammatory infiltration, the EB4 and EB4@L100‐55 groups exhibited preserved mucosal integrity and a significant reduction in inflammatory cell infiltration. This indicates that early intervention successfully prevented the typical inflammatory destruction of the gut architecture (Figure 7h and Figure S25). To elucidate the basis for this protection, we evaluated the intestinal retention capacity within this preventive context. Fluorescence scanning of colonic sections revealed prominent signals in the EB2, EB4, and EB4@L100‐55 groups (Figure 7i). Furthermore, plate counts of fecal samples collected daily prior to administration (Days 2–6) and at the final sacrifice (Day 7) confirmed that the EB4 and EB4@L100‐55 groups exhibited significantly higher retention titers, which is critical for inhibiting the onset and progression of IBD (Figure S26). We next investigated the capacity of the engineered probiotics to modulate oxidative stress and fortify the epithelial barrier. ROS fluorescence scanning and MPO activity assays demonstrated that the pathologically elevated ROS levels typically triggered by DSS were significantly suppressed by the engineered probiotics (Figures S27 and S28). Immunofluorescence staining for tight junction proteins demonstrated that while EB2, EB4, and EB4@L100‐55 all improved the distribution of Claudin and ZO‐1, only the platforms integrating the therapeutic IBD modules (EB4 and EB4@L100‐55) successfully promoted a continuous fluorescence signal for Occludin along the epithelial membrane. These results indicate that the synergistic action of ROS scavenging and mucosal repair reinforced cell‐cell junctions, thereby maintaining the integrity of the epithelial barrier during inflammatory stress (Figure S29). This suggests that the synergistic action of ROS clearance and mucosal repair successfully maintained the balance of the intestinal microenvironment and the integrity of the epithelial barrier during inflammatory stress. Finally, we analysed the colonic cytokine profile via ELISA (Figure 7j–q). Regarding pro‐inflammatory modulation, the engineered probiotic groups inhibited the elevation of IL‐1β, IL‐17A, IL‐6, TNF‐α, and IFN‐γ to varying degrees. Notably, while the anti‐inflammatory cytokines TGF‐β and IL‐10 showed slight increases in the Con and EB2 groups, these changes lacked statistical significance. In sharp contrast, EB4 and EB4@L100‐55 treatment resulted in significant upregulation of these anti‐inflammatory markers.

### 2.9 Gut Microbiota Modulation in The Prophylactic Model

**FIGURE 8:**
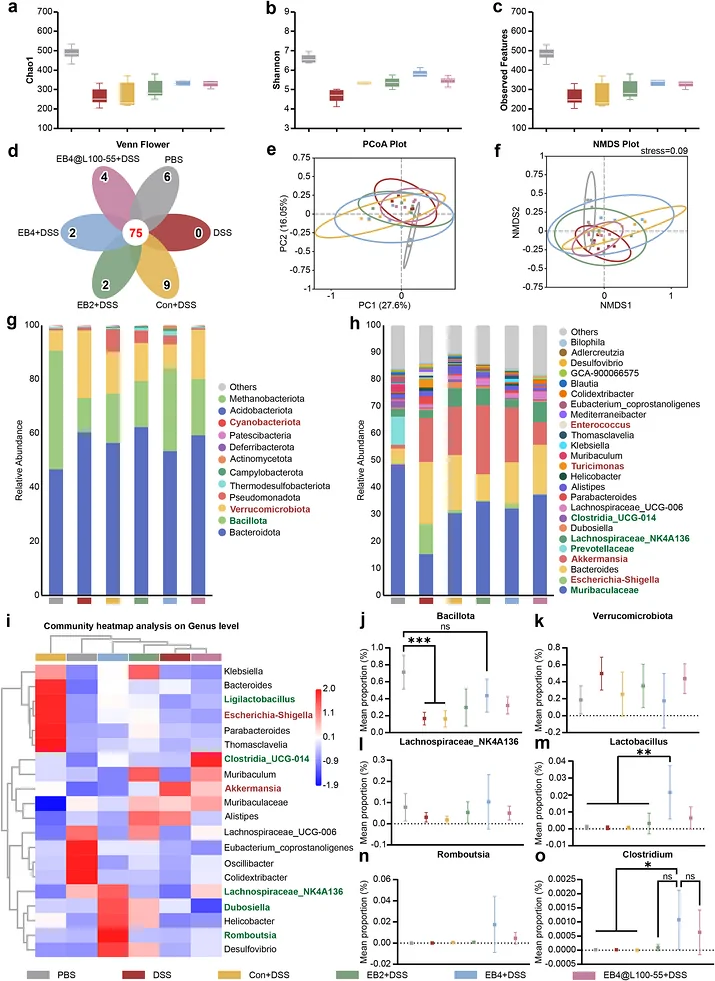
16S rRNA sequencing analysis of gut microbiota composition in the prophylactic model. (a–c) Microbial α‐diversity indices, including Chao1 (a), Shannon (b), and observed features (c), calculated for each experimental group. (d) Venn flower plot showing the distribution and overlap of operational taxonomic units (OTUs) across groups. (e,f) Microbial β‐diversity visualized by Principal Coordinate Analysis (PCoA) (e) and Non‐metric Multidimensional Scaling (NMDS) plot (f; stress = 0.09). Ellipses represent 95% confidence intervals. (g,h) Taxonomic composition of gut microbiota at the phylum level (g, top 10) and genus level (h, top 25). (i) Community heatmap analysis comparing the relative abundance of the top 20 genera. (j,k) Mean proportions of the phyla *Bacillota* (j) and *Verrucomicrobiota* (k). (l–o) Mean proportions of selected genus‐level taxa, including *Lachnospiraceae*_NK4A136 (l), *Lactobacillus* (m), *Romboutsia* (n), and *Clostridium* (o). Data are presented as mean ± SD derived from *n* = 5 biologically independent animals per group. Statistical significance was determined via one‐way ANOVA followed by Tukey's post‐hoc test (^***^ *p* < 0.001; ^**^ *p* < 0.01; ^*^ *p* < 0.05; ns, not significant). The names of key biologically relevant taxa are highlighted in bold, with anti‐inflammatory and beneficial taxa color coded in green, and pro‐inflammatory or pathobiont taxa marked in red.

To investigate the ecological impact of preemptive probiotic intervention on the gut microbiota, we performed 16S rRNA gene sequencing on colonic contents. Analysis of α‐diversity revealed that DSS‐induced stress precipitated a collapse in microbial richness, whereas the administration of EB4 and EB4@L100‐55 effectively counteracted this decline, as evidenced by the significant recovery in Chao1 and observed features indices (Figure 8a,c). Furthermore, Shannon and Simpson indices indicated that all engineered probiotic cohorts facilitated a general restoration of community evenness compared to the diseased group (Figure 8b and Figure S30). The distribution of OTUs visualized through a Venn diagram revealed that a shared healthy core consisting of 75 OTUs was maintained across all groups. Notably, the absence of unique OTUs in the DSS group, contrasted with the emergence of specific taxa in the probiotic‐treated cohorts, suggested that our engineered platforms promote the recruitment of beneficial species while preventing the establishment of a disease‐specific microbial signature (Figure 8d). Structural divergence was further characterized via β‐diversity analysis. PCoA, NMDS, and PCA plots indicated that DSS induction triggered an extensive drift in the microbial community architecture, resulting in a significant separation along the PC2 axis between the challenged groups and healthy controls (Figure 8e,f and Figure S31). Within this prophylactic window, the engineered probiotics demonstrated a discernible trend toward ecological stabilization, though the overall community shift remained substantial. Taxonomic profiling at the phylum level showed that DSS induction compromised the dominance of *Bacillota* and prompted an expansion of *Verrucomicrobiota* (Figure 8g). Remarkably, all engineered probiotic interventions effectively reversed these broad‐scale alterations, steering the microbial landscape toward a healthy configuration. At the genus level, DSS‐induced dysbiosis was characterized by a significant depletion of beneficial obligate anaerobes, including *Muribaculaceae*, *Prevotellaceae*, and *Clostridia*_UCG‐014. Simultaneously, we observed the pathological enrichment of pathobionts such as *Escherichia‐Shigella* and *Enterococcus*, alongside an abnormal elevation in the abundance of *Bacteroides* (Figure 8h). Notably, DSS treatment led to an expansion of *Verrucomicrobiota* (predominantly *Akkermansia*), while the engineered probiotic groups exhibited non‐linear fluctuations in its abundance. The engineered probiotics successfully recalibrated these aberrant microbial structures toward baseline states. Heatmap analysis of the top 20 genera provided higher resolution of these micro‐ecological changes (Figure 8i). Specifically, EB4 significantly increased the abundance of *Lachnospiraceae*_NK4A136, *Dubosiella*, and *Romboutsia*. In comparison, the EB4@L100‐55 group primarily promoted the abundance of *Lachnospiraceae*_NK4A136 and *Clostridia*_UCG‐014. Individual analysis confirmed that probiotic intervention fostered the recovery of *Bacteroidota* and suppressed *Verrucomicrobiota* (Figure 8j,k). Individual analysis of key taxa further elucidated the restorative effects of the engineered platforms on the colonic microbiota (Figure 8l–o). Probiotic intervention generally fostered the restoration of *Lachnospiraceae* NK4A136 across multiple engineered groups, although these shifts remained as non‐significant trends. Notably, the EB4 group demonstrated a superior capacity to enrich *Lactobacillus* compared to both the DSS and PBS groups. Similar elevations in mean abundance were observed for *Romboutsia* within the EB4 and EB4@L100‐55 groups, yet substantial intra group variance precluded formal statistical significance. Additionally, EB4 treatment specifically facilitated the recovery of *Clostridium* relative to the colitic controls. Finally, ternary plot analysis confirmed these distinct enrichment patterns, revealing that EB4@L100‐55 primarily increased the abundance of *Cyanobacteriota* at the phylum level, while *Ligilactobacillus* showed consistent enrichment across both the EB4 and EB4@L100‐55 groups (Figure S32).

Based on the results of the multiple group comparative analyses, the stabilizing effects of the engineered platforms on the intestinal microbiota were further elucidated (Figure S33). Prophylactic intervention fostered a general trend toward the restoration of *Lachnospiraceae*_NK4A136 across the engineered groups. Notably, the EcN‐EB4 strain demonstrated a significant enrichment of *Lactobacillus* in the prophylactic model. Furthermore, intervention with the engineered strains mitigated the aberrant pathological expansion of *Akkermansia* typically induced by DSS stress. Concurrently, the integrated platforms effectively maintained the suppression of *Escherichia‐Shigella*, thereby preventing the abnormal elevation observed following unmodified EcN‐Con administration. Collectively, these focused quantitative profiles suggest that preemptive administration of the engineered probiotics effectively buffers the intestinal microecology against inflammation driven structural collapse.

To further evaluate the functional metabolic changes in the prophylactic setting, the alterations in fecal butanoic acid were determined (Figure S34). DSS treatment induced a substantial loss of butanoic acid. In contrast, prophylactic intervention with the engineered strains effectively prevented this metabolic deterioration. Consistent with the enriched abundance of butyrate producing taxa such as *Lachnospiraceae*_NK4A136, both the EcN‐EB4 and EB4@L100‐55 strains demonstrated highly significant preservation of butanoic acid compared to the disease group. These results indicate that early intervention with the engineered platforms effectively sustains essential metabolic functions, functionally corroborating the restoration of the intestinal microbiota structure.

### 2.10 Modular Evaluation of the EB4@L100‐55 Formulation and Comparison With Standard Clinical Therapy

**FIGURE 9:**
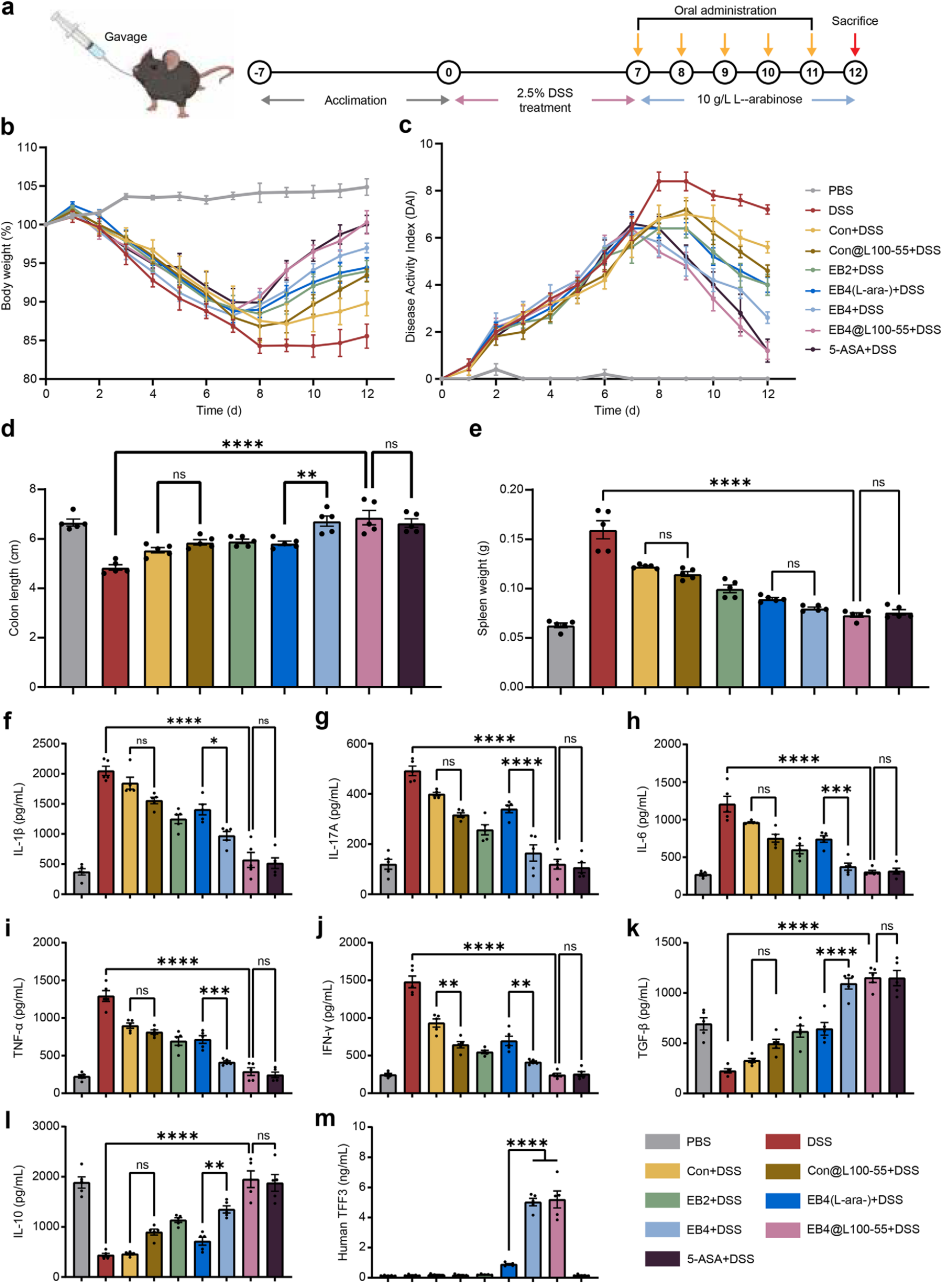
Modular evaluation of the EB4@L100‐55 formulation and comparison with standard clinical therapy in the delayed‐treatment colitis model. (a) Schematic representation of the DSS‐induced colitis induction and the delayed‐treatment protocol. Notably, the EB4(L‐ara‐)+DSS group received no L‐arabinose (L‐ara) during both the bacterial culture phase and the in vivo mouse intervention. (b, c) Body weight recovery trajectories (b) and disease activity index (DAI) scores (c) recorded over the 12‐day experimental period. (d, e) Statistical analysis of colon length (d) and spleen weight (e) from mice in the indicated experimental groups harvested on day 12. (f–l) ELISA quantification of pro‐inflammatory and anti‐inflammatory cytokines, including IL‐1β (f), IL‐17A (g), IL‐6 (h), TNF‐α (i), IFN‐γ (j), TGF‐β (k), and IL‐10 (l), in colon tissues. (m) ELISA quantification of secreted human TFF3 in colon tissues. Data are presented as mean ± SEM (*n* = 5 biologically independent animals per group). Statistical significance was determined via one‐way ANOVA followed by Tukey's post‐hoc test (^****^ *p* < 0.0001; ^***^ *p* < 0.001; ^**^ *p* < 0.01; ^*^ *p* < 0.05; ns, not significant).

To further delineate the specific contributions of individual engineered modules to the observed therapeutic efficacy, and to compare our platform against standard clinical treatments for colitis, we established an independent DSS‐induced cohort (Figure 9a). For this validation, we deliberately selected the delayed‐treatment paradigm rather than the prophylactic model, as it more accurately mimics real‐world clinical scenarios. Within this model, three additional groups were introduced and designated as Con@L100‐55+DSS, EB4(L‐ara‐)+DSS, and 5‐ASA+DSS. Specifically, Con@L100‐55+DSS was utilized to evaluate the inherent impact of the coating material; EB4(L‐ara‐)+DSS, an uninduced therapeutic strain, was employed to assess the necessity of mucosal anchoring; and 5‐ASA+DSS served as the clinical positive control.

First, we microencapsulated EcN‐Con with Eudragit L100‐55 to verify whether this coating material inherently exerted any immunomodulatory effects. Evaluation of macroscopic disease indicators revealed no significant differences in body weight recovery (Figure 9b), DAI score attenuation (Figure 9c), colon length (Figure 9d and Figure S35), or spleen weight (Figure 9e and Figure S36) between the uncoated EcN‐Con and the microencapsulated Con@L100‐55 groups. Histological evaluation via H&E staining further corroborated these macroscopic findings, as both the EcN‐Con and Con@L100‐55 groups exhibited persistent inflammatory cell infiltration and marked thickening of the bowel wall (Figure S37). Furthermore, the expression profiles of all evaluated pro‐inflammatory and anti‐inflammatory cytokines showed no significant statistical differences between these two groups (Figure 9f–l). This unequivocally confirms that the enteric shell acts exclusively as a delivery vehicle, and any subsequent disease alleviation is derived from the encapsulated core strains rather than the coating material itself.

Next, to determine the extent to which the therapeutic modules rely on mucosal anchoring, we evaluated the EB4(L‐ara‐) group, a strain carrying the complete therapeutic circuits but lacking the surface display of tSpaC. Strikingly, despite possessing identical ROS‐scavenging and TFF3‐secreting capabilities, the EB4(L‐ara‐) strain exhibited markedly inferior therapeutic outcomes. Compared to the fully induced EB4 group, EB4(L‐ara‐) failed to effectively restore colon length (Figure 9d and Figure S35) and demonstrated significantly weaker regulation of key cytokines (Figure 9f–l). Similarly, histopathological assessment revealed pronounced inflammatory infiltration in the colonic tissue of the EB4(L‐ara‐) group, whereas the fully induced EB4 treatment facilitated substantial restoration of the colonic mucosal architecture (Figure S37). This drastic attenuation provides definitive in vivo evidence that overcoming rapid gastrointestinal washout through bio‐interfacial anchoring and short‐term retention is a fundamental prerequisite for engineered probiotics to achieve effective local concentrations of therapeutic payloads.

Finally, we directly compared the therapeutic efficacy of EB4@L100‐55 with 5‐aminosalicylic acid (5‐ASA), a first‐line clinical drug for IBD. Notably, the EB4@L100‐55 platform achieved clinical remission comparable to that of 5‐ASA treatment across all evaluated parameters. The recovery trajectories of body weight and DAI scores (Figure 9b,c), the restoration of colon length (Figure 9d and Figure S35), and the comprehensive normalization of the systemic cytokine network (Figure 9f–l) showed no statistical differences between the EB4@L100‐55 and 5‐ASA groups. Furthermore, at the microscopic level, both the EB4@L100‐55 and 5‐ASA treatments exhibited excellent repair of the colonic tissue, effectively mitigating inflammatory infiltration and restoring epithelial integrity (Figure S37). Collectively, this mechanistic deconvolution and clinical comparison demonstrate that the final integrated strain, EB4@L100‐55, achieves comparable regulatory effects to existing standard‐of‐care drugs.

### 2.11 Systemic Biosafety Evaluation and Self‐Limiting Clearance Kinetics of EB4@L100‐55

To ensure the translational potential of our engineered platform, we comprehensively evaluated the in vivo biosafety of the final therapeutic formulation EB4@L100‐55. In healthy mice, continuous oral administration of EB4@L100‐55 did not induce any abnormal body weight fluctuations compared to the PBS control (Figure S38a). Moreover, histopathological analysis of major organs (heart, liver, spleen, lung, and kidney) and colonic tissues revealed no observable structural damage or inflammatory infiltration (Figure S38b). Crucially, we investigated the risk of bacterial translocation in the DSS‐induced colitis model. H&E staining of the extra‐intestinal organs from colitic mice treated with EB4@L100‐55 showed no pathological lesions (Figure S38c). Notably, while the PBS‐treated colitic mice exhibited persistent local inflammatory infiltration in the colon, the colonic tissues of the EB4@L100‐55‐treated group demonstrated substantial restoration of the mucosal architecture. To definitively rule out translocation, we quantified the bacterial loads in the tissue homogenates of the liver, spleen, and kidney from both healthy and colitic cohorts via plate colony counting. Strikingly, no viable engineered bacteria were detected in any of the extra‐intestinal organs, confirming that our tSpaC‐displaying platform does not induce pathogenic translocation.

Finally, we plotted the bacterial clearance curves using an independent delayed‐treatment DSS cohort to track the in vivo retention dynamics of the engineered strains (Figure S39). In this model, mice were treated with 2.5% DSS from day 0 to day 7 to induce colitis, followed by a 5‐day daily gavage of probiotics from day 7 to day 11. Fecal bacterial loads were continuously monitored from day 7 until complete clearance was achieved. The control strains lacking functional adhesins experienced rapid washout; specifically, EcN‐Con and the uninduced EcN‐EB4(L‐ara‐) were entirely cleared by Day 15. The microencapsulated control, Con@L100‐55, exhibited a slight increase in the in vivo abundance but only marginally prolonged the retention time to Day 16. In contrast, the tSpaC‐displaying strains exhibited significantly extended intestinal retention. Interestingly, a divergent clearance pattern emerged among these adhesion‐enhanced cohorts: the non‐therapeutic EcN‐EB2 strain anchored persistently up to Day 20, whereas both therapeutic strains, EcN‐EB4 and EB4@L100‐55, were completely cleared earlier by Day 17. This dynamic divergence aligns with our histological and ecological findings, suggesting that the mucosal repair orchestrated by the therapeutic strains effectively restores the epithelial barrier, thereby diminishing available binding sites, while simultaneously promoting the recovery of colonization resistance by the native microbiota. Collectively, these synergistic factors drive the accelerated, self‐limiting clearance of the engineered probiotics upon disease remission.

To quantitatively characterize post‐treatment bacterial clearance, fecal bacterial loads during the clearance phase were fitted using a single‐exponential decay model. The non‐therapeutic adhesion‐enhanced strain EcN‐EB2 exhibited a clearance rate constant of 0.447 d^−1^ (95% CI: 0.354–0.561) and an estimated half‐life of 1.550 d (95% CI: 1.235–1.956). In comparison, EcN‐EB4 and EB4@L100‐55 exhibited higher estimated clearance rate constants of 0.961 d^−1^ (95% CI: 0.799–1.167) and 1.083 d^−1^ (95% CI: 0.912–1.299), respectively, with corresponding half‐lives of 0.721 d (95% CI: 0.594–0.868) and 0.640 d (95% CI: 0.533–0.760). These clearance rate constants were approximately 2.15‐ and 2.42‐fold higher than that of EcN‐EB2, respectively, quantitatively supporting the faster self‐limiting clearance of the therapeutic strains following treatment cessation (Figure S39b,c).

## 3 Discussion

The clinical translation of LBPs for inflammatory bowel disease is often compromised by their rapid clearance from the gastrointestinal tract. Here, we present the EB4@L100‐55 platform that addresses this limitation through the integration of a genetically enhanced, adhesion‐optimized chassis with an inflammation‐responsive therapeutic circuit. A central achievement of this study is the functional display of the Gram‐positive *L. rhamnosus* GG‐derived SpaC adhesin on a Gram‐negative *E. coli* Nissle 1917 host. While previous strategies have achieved enhanced intestinal persistence using adhesins from closely related species [37, 38], our approach successfully bridges the evolutionary divide between distinct bacterial secretion systems, demonstrating that complex, cross‐species surface display engineering is feasible through systematic structural optimization. Despite extensive advances in biocatalysis and drug delivery, a universal surface display system suitable for all proteins has yet to be developed [24, 39, 40, 41, 42]. The application of these systems remains constrained by the intrinsic properties of the target proteins, including molecular weight and specific folding requirements. While non‐genetic methods involving the conjugation of antibodies onto bacterial surfaces can bypass certain technical hurdles, such strategies fail to confer a retention advantage to daughter cells during bacterial proliferation [43]. In contrast, our genetic engineering approach ensures stable, multi‐generational inheritance of the enhanced adhesion phenotype. By combining surface display system screening, the substitution of constitutive promoters with inducible ones, and protein truncation, we ensured both plasmid stability and successful protein expression. This integrated strategy offers a valuable methodological framework for future surface display engineering. Furthermore, the superior intestinal retention efficacy of EB4@L100‐55 stems from the multi‐valent binding capacity of the truncated SpaC adhesin. In contrast to single‐target strategies such as the LAP‐Hsp60 interaction, SpaC simultaneously recognizes both the intestinal mucus layer and the extracellular matrix components exposed during mucosal injury [16, 17, 18]. This bifunctional anchoring mechanism ensures resilient retention even within the severely compromised epithelial landscapes characteristic of active IBD.

The distinct physical saturation thresholds observed in our ex vivo adhesion models provide direct mechanistic insights into this disease‐targeted enrichment of the tSpaC‐displaying platform. In the healthy colonic tissue, adhesion reached a saturation plateau at a dose of 1 × 10^6^ CFU. This early saturation reflects the anatomical integrity of the healthy intestine, where an intact mucus layer serves as a physical barrier, limiting the physiological binding sites accessible to the engineered bacteria. Conversely, the DSS‐induced inflamed colon required a higher concentration, reaching maximum saturation at 1 × 10^7^ CFU. This expanded binding capacity confirms that the pathological depletion of mucin and the subsequent exposure of the extracellular matrix in ulcerated lesions provide additional binding targets for tSpaC, facilitating specific bio‐interfacial accumulation at sites of inflammation. While these ex vivo assays indicate 1 × 10^7^ CFU as the saturation capacity for inflamed tissues, translating this finding into an effective in vivo dosing regimen requires consideration of the dynamic gastrointestinal environment. Oral administration is subject to physiological clearance mechanisms, including mechanical shear forces from peristalsis, enzymatic degradation, and the diarrheal washout characteristic of inflammatory bowel disease. These factors can cause a substantial reduction in viable bacteria before they reach the colonic mucosa. As demonstrated by the dose‐dependent data, if the localized effective concentration decreases to 1 × 10^6^ CFU or lower, therapeutic adhesion on inflamed lesions is compromised. To compensate for these dynamic losses and maintain the final bio‐interfacial concentration near the 1 × 10^7^ CFU saturation capacity, administering an initial dose of 1 × 10^9^ CFU was deemed appropriate. This selected dose provides a safety margin to secure therapeutic coverage while avoiding the potential metabolic burden associated with higher doses such as 1 × 10^10^ CFU.

The therapeutic efficacy of the EB4@L100‐55 platform is further augmented by a closed‐loop circuit that synchronizes disease sensing with multi‐pronged intervention. While previously reported sensors for transient small molecules like thiosulfate or nitric oxide offer rapid responses, they often suffer from localized signal fluctuations and limited clinical correlation [44, 45]. In contrast, our platform utilizes the endogenous *ykgMO* promoter to sense calprotectin [19], which is a clinical gold standard biomarker for IBD severity [20, 46]. Our system demonstrates precise induction within the clinically relevant range of 0 to 800 µg/mL, ensuring that therapeutic output is quantitatively calibrated to the degree of intestinal inflammation [47, 48]. This autonomous sensing strategy minimizes off‐target metabolic costs while maximizing localized drug concentration during flare‐ups. Upon activation, the circuit initiates a coordinated response between suppressing inflammatory drivers and restoring mucosal integrity. Secretion of TFF3 via a YebF‐mediated carrier is central to achieving epithelial restitution, a fundamental clinical endpoint in IBD management that prevents luminal antigen influx [49]. Unlike matrix‐tethered strategies such as CsgA‐TFFs [9], this secretory approach enables TFF3 to efficiently diffuse into the compromised epithelial interface to accelerate structural restitution. Thus, this integrated design concurrently addresses both the chemical mediators of inflammation and the physical disruption of the epithelial barrier.

Existing antioxidant microbial therapies typically rely on single‐enzyme or dual‐enzyme systems such as CAT‐SOD [10, 50]. The canonical CAT‐SOD‐GPx antioxidant cascade has been described as a sequential and complementary ROS‐detoxification mechanism, in which SOD converts superoxide anions into hydrogen peroxide and CAT and GPx subsequently remove hydrogen peroxide [51, 52]. Our platform incorporates these three protein components into a CAT‐SOD‐GPx tripartite fusion system. However, the fused GPx exhibited no discernible catalytic activity under the present in vitro conditions. Experimentally, a notable finding of our engineering strategy was that incorporation of GPx markedly enhanced the soluble expression of the CAT‐SOD fusion and reduced aggregate inclusion body formation (Figure 3d), accompanied by increased CAT and SOD activities (Figure 3e,f). These experimentally observed effects indicate a structural contribution of GPx to the fusion construct but do not establish its underlying mechanism. We therefore hypothesize that GPx may function as a non‐catalytic structural stabilizer or chaperone‐like fusion partner. In this proposed model, GPx might act as a thermodynamic stabilizer that lowers the free‐energy barrier of folding intermediates and masks hydrophobic patches prone to aggregation. By functioning similarly to a solubilizing tag, this putative chaperone‐like activity could explain the improved solubility of the fusion construct. This design principle offers a novel framework for assembling complex synthetic biological modules within living therapeutics, though future structural assays will be required to confirm this mechanism.

**FIGURE 10:**
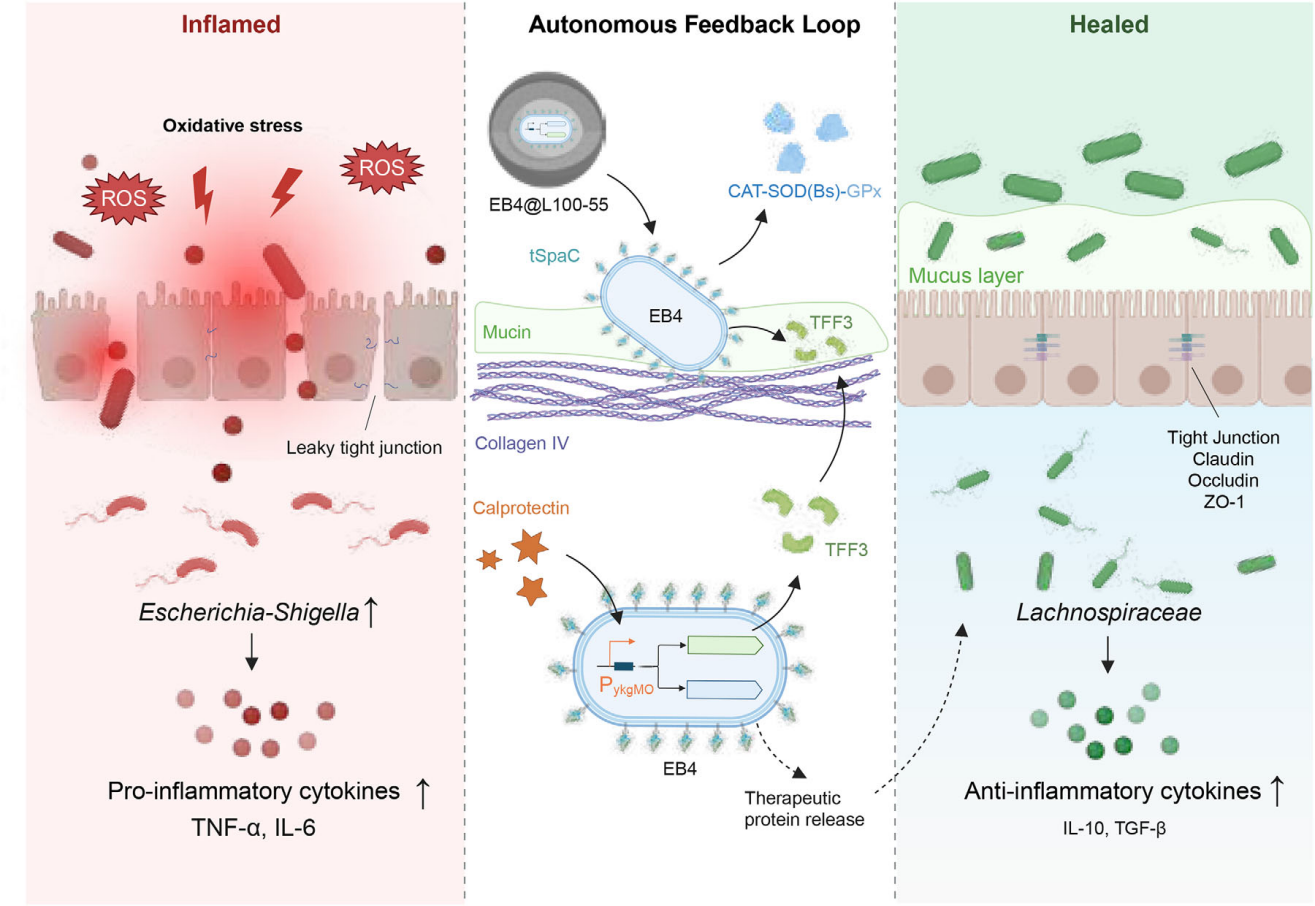
Schematic representation of the therapeutic mechanisms orchestrated by the EB4@L100‐55 platform. Upon oral administration, the Eudragit L100‐55 coating ensures the pH‐responsive release of engineered EcN (EB4) in the distal intestine. The surface‐displayed tSpaC mediates robust and sustained anchoring to intestinal mucins and exposed extracellular matrix components. At inflamed sites, the sensing of pathological calprotectin triggers the autonomous production of the CAT‐SOD‐GPx TFS to scavenge ROS and the secretion of TFF3 to promote epithelial restitution. This multidimensional intervention leads to a systemic recalibration of the colonic microenvironment, characterized by: (i) Microbiological restoration, where engineered EcN outcompetes opportunistic pathobionts (e.g., *Escherichia‐Shigella*) via niche competition while promoting the resurgence of butyrate‐producing beneficial taxa (e.g., *Lachnospiraceae*); (ii) Immunological rebalancing, evidenced by the suppression of pro‐inflammatory cytokines (e.g., TNF‐α, IL‐6) and the upregulation of anti‐inflammatory mediators (e.g., IL‐10, TGF‐β); and (iii) Barrier fortification, through the enhanced expression of tight junction proteins. Created with BioRender.com.

The therapeutic efficacy of our platform stems from a systemic recalibration of the colonic microenvironment, coordinating molecular intervention with ecological restoration (Figure 10). IBD pathogenesis is inextricably linked to gut microbial dysbiosis, a state that exacerbates mucosal inflammation and barrier dysfunction [53, 54, 55]. Beyond direct immunomodulation, our engineered probiotics facilitated a profound restructuring of the gut microbiota by reversing the depletion of *Bacteroidota* and *Bacillota*. These phyla are essential for complex polysaccharide fermentation and the production of short‐chain fatty acids (SCFAs) [56, 57]. A compelling experimental observation in our models was the significant suppression of opportunistic pathobionts, notably *Escherichia‐Shigella* and *Enterococcus* [58, 59, 60, 61, 62]. Given the close taxonomic relationship between the EcN chassis and *Escherichia‐Shigella*, we propose that the reduction in *Escherichia‐Shigella* may partly reflect niche competition [63], whereas the concurrent decrease in *Enterococcus* may arise indirectly from broader remodeling of the colonic microenvironment. Although direct competitive exclusion assays were not performed in this study, we hypothesize that the enhanced tSpaC‐mediated anchoring provides the engineered EcN with a critical spatial advantage, enabling it to outcompete pathogenic relatives for limited mucosal attachment sites and nutrients. This competitive exclusion, coupled with oxidative stress mitigation, guides the intestinal environment from a diseased state toward stable microbial homeostasis.

Furthermore, the recovery of beneficial obligate anaerobes such as *Muribaculaceae* and *Lachnospiraceae* underscores the holistic nature of this recovery. Members of the *Lachnospiraceae* family, including *Colidextribacter* and *Lachnospiraceae*_NK4A136, are known for producing butyrate, which serves as a primary energy source for colonocytes and promotes T‐regulatory cell differentiation to support epithelial health [64, 65, 66, 67]. Similarly, enrichment of *Dubosiella* and *Romboutsia* provides critical support for the restoration of intestinal barrier integrity. *Dubosiella* has been increasingly recognized for its role in modulating immune homeostasis and metabolic health [68, 69], while *Romboutsia* is associated with epithelial stability and robust metabolic activity [70, 71]. The non‐linear fluctuations of *Verrucomicrobiota*, predominantly *Akkermansia*, represent a particularly noteworthy ecological transition. While its expansion in the disease group likely indicates a compensatory response to the accelerated shedding of the mucus layer, its dynamic shift in treated groups signifies a state of active microbial succession [72, 73]. The irregularity reflects the complex recalibration occurring as TFF3‐mediated repair and ROS scavenging stabilize the mucosal niche. Collectively, the robust physiological remission and beneficial shifts in microbial architecture underscore the translational potential of this programmable probiotic platform for precision management of acute colonic inflammation.

Despite the robust performance of the EB4@L100‐55 platform, several limitations warrant further investigation. The current system relies on plasmid‐based expression for the therapeutic modules, which carries the potential risk of plasmid loss in the absence of antibiotic selection pressure within the complex gut environment. Although our study demonstrated significant efficacy in murine models, the genomic integration of these functional circuits will be necessary to ensure long‐term genetic stability and prevent horizontal gene transfer. Furthermore, the precise biophysical mechanism by which GPx acts as a structural chaperone to facilitate the soluble expression of the TFS construct remains to be fully elucidated. Future studies employing high‐resolution structural analysis or molecular dynamics simulations are required to explore the folding landscape of this tripartite fusion construct. Additionally, transition from murine colitis models to human clinical applications must account for the substantially different microbial diversity and transit kinetics of the human gastrointestinal tract. In this context, translational prospects must also be balanced against regulatory challenges and potential risks, particularly regarding long‐term safety, bacterial clearance kinetics, and possible bacterial translocation. Our current study rigorously evaluated the safety and clearance profiles of the engineered bacteria in both healthy and inflammatory models. The existing results demonstrate rapid elimination in healthy hosts, self‐limiting clearance in colitic environments, and a complete absence of bacterial translocation to major organs. However, these findings are primarily derived from single acute colitis models. For chronic inflammatory conditions, long‐term intervention protocols using engineered probiotics require strictly prolonged evaluation. To guarantee absolute biosafety and ensure rapid bacterial clearance upon treatment completion, future development of engineered probiotics will necessitate the incorporation of robust biocontainment mechanisms, such as environment‐responsive kill switches.

Despite these remaining safety concerns regarding clinical translation, this study establishes a significant engineering paradigm for the development of high‐performance LBPs. By integrating cross‐species surface display, inflammation‐responsive logic, and a TFS‐based antioxidant module, we have demonstrated a successful strategy to overcome the primary bottlenecks of microbial therapy. This programmable probiotic platform holds substantial potential for the precision management of acute intestinal inflammation. Importantly, this work provides critical insights into how such programmable platforms can effectively alleviate inflammation during its active state. Future research will focus on evaluating these systems in chronic colitis models, as well as the genomic stabilization of these functional modules and the assessment of their long‐term ecological impact in diverse host environments to facilitate clinical translation.

## 4 Experimental Section

### 4.1 Bacterial Strains, Plasmids, and Genetic Engineering

The experimental framework involved the construction of an adhesion‐enhanced EcN chassis via surface display of a truncated SpaC (tSpaC) adhesin, followed by the integration of biomarker‐responsive therapeutic modules. Bacterial strains and plasmids utilized in this study are detailed in Table S1. *E. coli* DH10B and BL21(DE3) were employed for molecular cloning and recombinant protein production, respectively. EcN (BNCC361741, BeNa Culture Collection, China) served as the therapeutic chassis.

Genetic circuits were assembled using Gibson assembly or whole‐plasmid PCR (Figure S40). The surface display vector, pET‐araBAD‐tSpaC, was engineered by fusing the codon‐optimized tSpaC sequence downstream of the INPN domain and the pelB signal peptide. For the antioxidant module, a CAT‐SOD‐GPx tripartite fusion construct was generated: the *Lactobacillus plantarum* catalase (CAT) gene was linked to superoxide dismutases (SOD) from *Lactococcus lactis* or *Bacillus subtilis* via an A(EAAAK)_3_A rigid linker, followed by the incorporation of *Lactobacillus casei* glutathione peroxidase (GPx) using a (GGGS)_3_ flexible linker. The entire cassette, along with the *yebF‐TFF3* secretion module, was placed under the regulation of the calprotectin‐responsive *ykgMO* promoter to generate the final therapeutic construct, pET‐tSpaC‐mGFP‐TFS‐TFF3. All primer sequences are provided in Table S2.

### 4.2 Protein Expression, Purification, and In Vitro Sensing

Recombinant protein expression was evaluated via SDS‐PAGE and Western blot analysis. Briefly, cultures were induced with 0.25% L‐(+)‐arabinose or recombinant calprotectin and lysed by sonication. Next, proteins were resolved by SDS‐PAGE and subsequently transferred onto PVDF membranes. Membranes were probed with anti‐His or anti‐3×Flag primary antibodies and visualized using the Odyssey CLx imaging system. For dose‐response characterization, the *ykgMO* promoter activity was quantified using mNeonGreen (mGFP, GenBank: EU482389) reporter system. EcN‐ykgMO‐mGFP was exposed to purified recombinant calprotectin (0–800 µg/mL), with fluorescence (Ex/Em: 485/530 nm) monitored via a TECAN SPARK reader. Recombinant calprotectin (GenBank: CR407674.1 for S100A8, CR542224.1 for S100A9) was purified from *E. coli* BL21(DE3)‐S100A8/A9 using Ni‐NTA affinity chromatography followed by buffer exchange into storage buffer (20 mM Tris‐HCl, 150 mM NaCl, pH 7.4).

### 4.3 Bio‐Interfacial Adhesion and Bioactivity Assays

The bio‐interfacial anchoring of engineered EcN was quantified using mucin‐ and collagen‐coated Nunc MaxiSorp plates. Adherent bacteria (1 × 10^6^ CFU input) were recovered after 3 h of incubation at 37°C and quantified by serial dilution plating. For cellular adhesion, Caco‐2 monolayers (ATCC #HTB‐37) were utilized; surface‐displayed tSpaC was visualized via immunofluorescence staining with anti‐His primary and YSFluor488‐conjugated secondary antibodies using a Zeiss LSM 900 confocal microscope.

The individual CAT, SOD, and GPx activities associated with the fusion constructs were assessed using CAT (R21885, Yuanye), SOD (R33183, Yuanye), and GPx (R33187, Yuanye) activity kits, respectively. Total antioxidant capacity (TAC) was further validated using ABTS (S0121, Beyotime) and FRAP (S0116, Beyotime) assays. Secreted TFF3 levels were quantified via ELISA (Elabscience) and normalized to total protein content.

### 4.4 Ex Vivo Colonic Adhesion Assay

To evaluate the bio‐interfacial anchoring capacity and determine the optimal binding dosage on native tissues, ex vivo colonic adhesion assays were performed. Briefly, mice were fasted for 24 h prior to sacrifice to empty the bowel. Colonic tissues were then excised from both healthy and DSS‐treated C57BL/6 mice and gently rinsed with cold PBS to remove residual fecal contents in the lumen. Subsequently, the tissues were co‐incubated with various concentrations of engineered probiotics at 37°C for 3 h. Following incubation, the tissues were washed with PBS to remove unadhered bacterial strains. Finally, the tissues were transferred to a transparent culture dish, and the fluorescence signals of the stably adherent bacteria were acquired and quantified as total radiant efficiency utilizing an in vivo imaging system.

### 4.5 Enteric Microencapsulation and Gastrointestinal Survival

To ensure gastric bypass, EcN‐EB4 was encapsulated with Eudragit L100‐55. Briefly, bacteria were incubated with 12.5 mM CaCl_2_ followed by the addition of Eudragit L100‐55 (40 µg/mL) at pH 5.0 [35]. The resulting EB4@L100‐55 was characterized by TEM (ImageJ for size distribution), dynamic light scattering (DLS), and zeta potential analysis. Survival rates were determined by exposing encapsulated and naked bacteria (1 × 10^9^ CFU) to simulated gastric fluid (SGF, pH 2.0) and simulated intestinal fluid (SIF, pH 7.0) for 1–2 h.

### 4.6 Flow Cytometric Analysis of tSpaC Surface Display and Gastrointestinal Stability

To quantify the surface display efficiency of tSpaC and its structural stability, flow cytometric analysis was performed. Samples of the indicated engineered strains and the microencapsulated formulation (EB4@L100‐55) were collected prior to digestion (Untreated), following 2 h of incubation in SGF, and after an additional 2 h of sequential incubation in SIF. Additionally, the wild‐type strain (EcN‐WT) was analyzed as a negative control to determine basal background fluorescence. The collected cells were washed twice with PBS via centrifugation. For immunofluorescent labeling, the bacterial suspensions were incubated with a PerCP/Cyanine5.5‐conjugated 6*His, His‐Tag Monoclonal antibody (Cat No. 362622, BioLegend) in the dark. Following washes with PBS to remove unbound antibodies, the samples were analyzed using a BD FACSCanto II flow cytometer (BD Biosciences). The gating strategy was established according to standard flow cytometric protocols. Initially, the intact bacterial population was gated based on forward scatter (FSC‐A) and side scatter (SSC‐A) profiles to exclude debris. Given the constitutive mGFP expression of the engineered strains, successful surface display was characterized by mGFP and PerCP/Cyanine5.5 dual fluorescence. Accordingly, the tSpaC‐positive rate was quantified as the percentage of these dual‐positive (FITC+/PerCP‐Cy5‐5‐A+) events within the overall mGFP‐gated population.

### 4.7 In Vivo Evaluation in Murine Colitis Models

All animal procedures were approved by the Institutional Animal Care and Use Committee (Approval No. DRK‐202507240331). Male C57BL/6 mice (6–8 weeks) were subjected to DSS‐induced colitis (2.5% w/v DSS in drinking water). Intervention groups received daily oral gavage of 1 × 10^9^ CFU of engineered EcN strains. Disease activity index (DAI) was recorded based on weight loss, stool consistency, and fecal occult blood (Table S3). Intestinal permeability was assessed via the FITC‐dextran (FD4) translocation assay. Post‐euthanasia, colon segments were processed for H&E staining and immunofluorescence (ZO‐1, Occludin, and Claudin). Microbiota profiling was performed by 16S rRNA gene sequencing (V3‐V4 region) on the Illumina platform, with bioinformatic analysis implemented via QIIME 2 and the UCHIME algorithm.

### 4.8 Statistical Analysis

Data are presented as mean ± SEM (or mean ± SD where indicated) from at least three independent biological replicates. Statistical comparisons between two groups were performed using an unpaired two‐tailed Student's t‐test. For multiple group comparisons, one‐way or two‐way ANOVA followed by Tukey's or Sidak's post‐hoc tests was employed. Statistical significance was defined as *p* < 0.05. All analyses were conducted using GraphPad Prism 10. Significant levels are denoted as: ^****^ *p* < 0.0001; ^***^ *p* < 0.001; ^**^ *p* < 0.01; ^*^ *p* < 0.05; ns (*p* ≥ 0.05).

## Author Contributions

Y.‐X.W. and Q.‐H.C. designed the experiments. Y.‐X.W., P.‐C.Z., S.‐M.G., N.‐N.J., and J.‐R.Z. prepared the engineered probiotics and performed in vitro experiments. Y.‐X.W. and X.‐Y.L. performed the animal experiments and analysed the data. Y.‐X.W., H.‐Y.L., X.‐Y.L., P.‐C.Z., S.‐M.G., N.‐N.J., J.‐R.Z., E.‐B.X., Y.S., and Q.‐H.C. wrote the paper. All authors discussed the experimental results and edited the manuscript.

## Conflicts of Interest

The authors declare no conflicts of interest.

## Supporting information




**Supporting File**: advs77743‐sup‐0001‐SuppMat.docx.

## Data Availability

The 16S rRNA sequencing data generated in this study are deposited in the NCBI Sequence Read Archive under accession number PRJNA1429807. The authors declare that all other data supporting the findings of this study are within the article and its Supporting Information file. Source data are provided with this paper.
