# Supplementary material for: Adhesion‐Enhanced Engineered Probiotic for Prolonged Intestinal Retention and Calprotectin‐Responsive IBD Therapy
Source: Adv Sci (Weinh). 2026 Sep 11:e77743. Online ahead of print. doi: 10.1002/advs.77743 (PMC13564191; doi:10.1002/advs.77743)
Supplement: Supplementary file 1 [file ADVS-9999-e77743-s001.docx]

Supporting Information

Adhesion-Enhanced Engineered Probiotic for Prolonged Intestinal Retention and Calprotectin-Responsive IBD Therapy

Yuxi Wang^1,2^, Peichao Zhang^2^, Simin Gu^1^, Hongyun Lu^3^, Ningning Jiang^2^, Jiarun Zhao^2^, Enbo Xu^1,2^, Xiayu Liu^2^*, Ying Shi^1^*, Qihe Chen^1,2,4^*

^1^ Department of Food Science and Nutrition, Zhejiang University, Hangzhou, China.

^2^ Future Food Laboratory, Innovation Centre of Yangtze River Delta, Zhejiang University, Jiaxing, China.

^3^ School of Life and Health Sciences, Hubei University of Technology, Wuhan, China.

^4^ Zhejiang Key Laboratory of Functional Structural Lipid Synthesis and Application, Jiashan, China.

*Corresponding author. [chenqh@zju.edu.cn](mailto:chenqh@zju.edu.cn); [shiying0520@zju.edu.cn](mailto:shiying0520@zju.edu.cn); [xiayuliu@zju.edu.cn](mailto:xiayuliu@zju.edu.cn)

**This Supporting Information file includes:**

Figs. S1 to S40

Tables S1 to S3

**Figure S1. SDS-PAGE analysis of tSpaC expression and subcellular localization.**

SDS-PAGE gel image showing the expression and distribution of tSpaC protein across different surface display systems in the EcN chassis. All cultures were grown at 37 °C and induced with 0.2% L-(+)-arabinose at an OD600 of 0.4 to 0.6. Lane M represents the protein molecular weight marker. The experimental groups are categorized into the empty vector control, ClyA-tSpaC, INPN-tSpaC, and AIDA-tSpaC. For each group, cell lysates were fractionated into the supernatant (S) and the pellet (P). The theoretical molecular weight of the INPN-tSpaC fusion protein is 74.4 kDa.

**Figure S2. Growth kinetics of EcN-EB2 under varied induction conditions.**

All bacterial strains were incubated at 37 °C. For the induced group (EcN-EB2 + L-ara), L-arabinose was added to a final concentration of 0.2% when the OD_600_ reached 0.4 to 0.6. EcN-Con and non-induced EcN-EB2 (EcN-EB2 - L-ara) served as controls. OD_600_ values were monitored over a 16-hour period. Data are presented as mean ± SEM (n = 3 independent experiments).

**Figure S3. Adhesion of EcN strains to Caco-2 cell monolayers.**

Adhesion capacities of EcN-EB2 and EcN-Con were evaluated using a Caco-2 cell monolayer model. Fluorescence images were captured via inverted fluorescence microscopy, and the mean fluorescence intensity (mGFP, arbitrary units) was quantified from six randomly selected fields per group. Data are presented as mean ± SEM (n = 6 independent biological replicates). Statistical significance was determined using a two-tailed Student’s t-test (****p < 0.0001).

**Figure S4. *In vivo* intestinal retention kinetics of EcN-Con and EcN-EB2 in mice.**

Strains were orally administered once daily for five consecutive days from Day 0 to Day 4. Fecal samples were collected daily, and bacterial loads were quantified via plate colony counting. Fecal EcN counts are expressed as Log_10_ CFU/g. Data are presented as mean ± SEM (n = 3 biologically independent animals per group).

**Figure S5. *Ex viv*o evaluation of tSpaC-mediated colonic adhesion.**

(a, b) Total radiant efficiency of the control strain EcN-Con and the adhesion-enhanced strain EcN-EB2 co-incubated with healthy colonic tissues. (a) Direct comparison between EcN-Con and EcN-EB2 at 1 × 10^9^ CFU. (b) Dose-dependent adhesion of EcN-EB2 across a concentration gradient from 1 ×10^5^ to 1 × 10^10^ CFU. (c, d) Total radiant efficiency of strains co-incubated with DSS-induced inflamed colonic tissues, showing the comparison at 1 × 10^9^ CFU (c), and the dose-dependent adhesion gradient of EcN-EB2 (d). Data are presented as mean ± SEM (n = 3 biologically independent animals). Statistical significance was determined via two-tailed Student’s t-test (a, c) or one-way ANOVA followed by Tukey’s post-hoc test (b, d) (***p < 0.001; *p < 0.05; ns, not significant).

**Figure S6. Safety evaluation of EcN-EB2 via body weight monitoring in healthy mice.**

Healthy mice were orally administered 1×10^9^ CFU of engineered EcN-EB2 or an equal volume of PBS buffer (PBS-Control) once daily for seven consecutive days. Body weight was monitored daily and is expressed as a percentage of the initial weight on Day 0. Data are presented as mean ± SEM (n = 5 biologically independent animals per group). Statistical significance between groups over time was determined using a two-way ANOVA followed by Sidak's multiple comparisons test.

**Figure S7. Hematological and biochemical parameters of healthy mice after oral administration of EcN-EB2.**

Blood samples were collected from healthy mice after seven consecutive days of oral administration of 1×10^9^ CFU EcN-EB2 or PBS (Control). Hematological indices, including white blood cell (WBC) count, red blood cell (RBC) count, platelets (PLT), neutrophils (Neut), lymphocytes (Lymph), monocytes (Mono), hematocrit (HCT), mean corpuscular volume (MCV), and mean corpuscular hemoglobin (MCH), were measured. Biochemical parameters, including alanine aminotransferase (ALT), aspartate aminotransferase (AST), albumin (ALB), alkaline phosphatase (ALP), total protein (TP), blood urea nitrogen (BUN), and creatinine (CREA), were quantified (n = 5 independent biological replicates). Statistical significance was determined using a two-tailed Student’s t-test.

**Figure S8. Histological analysis of major organs following oral administration of EcN-EB2.**

Representative H&E staining images of the colon, lung, liver, spleen, kidney, and heart from healthy mice are presented. Tissue samples were collected after seven consecutive days of oral administration of 1×10^9^ CFU EcN-EB2 or PBS (Control). Scale bar represents 200 μm.

**Figure S9. Expression and purification of recombinant calprotectin in *E. coli* BL21(DE3).**

Expression of S100A8 and S100A9 proteins was analyzed via (a) SDS-PAGE and (b) Western blot. *E. coli* BL21(DE3) cells harboring either the empty vector (EV) or the S100A8/A9 expression plasmid were cultured in Terrific Broth (TB) at 37 °C. Protein expression was induced with 0.5 mM IPTG at an OD_600_ of 0.4 to 0.6, followed by incubation at 20 °C for 24 h. Lane M represents the protein molecular weight marker. Cell lysates were fractionated into supernatant (S) and pellet (P) components. Lane PP denotes the purified recombinant calprotectin protein.

**Figure S10. Quantitative analysis of tSpaC surface display and in vitro gastrointestinal stability.**

The tSpaC-positive rates of the indicated engineered strains (EcN-Con, EcN-EB2, EcN-EB4) and the microencapsulated formulation (EB4@L100-55) were determined via flow cytometry. To assess the stringency of the inducible system, EcN-EB4(L-ara-) was cultured in the absence of L-arabinose as an uninduced control. Samples were analyzed at three sequential stages: prior to digestion (Untreated), following incubation in simulated gastric fluid (SGF), and after subsequent incubation in simulated intestinal fluid (SGF+SIF). Data are presented as individual biological replicates (n = 3 independent biological replicates), with horizontal lines indicating the mean.

**Figure S11. *Ex vivo* fluorescence imaging of the gastrointestinal tracts in the colitis model.**

Representative images of the stomach and intestinal tissues harvested 4 h after a single oral gavage in mice with DSS induced colitis. (a) PBS group, (b) EcN-Con group, (c) EcN-EB4 group, and (d) EB4@L100-55 group. The pseudocolor heatmaps represent the radiant efficiency (p/sec/cm^2^/sr) of the mGFP expressing probiotics, illustrating the spatial distribution and accumulation of the bacteria within the inflamed intestinal regions.

**Figure S12. Quantitative analysis of the intestinal retention efficiency.**

Total radiant efficiency of the bacterial fluorescence in the colonic tissues was quantified 4 h post administration for the indicated groups. Data are presented as mean ± SEM (n = 4 biologically independent animals per group). Statistical significance was determined by one-way ANOVA with Tukey’s post hoc test. **p < 0.01; ns, not significant.

**Figure S13. Representative images of mouse spleens in the delayed treatment model.**

Spleens were harvested from mice across the indicated experimental groups, including PBS, DSS, Con+DSS, EB2+DSS, EB4+DSS, and EB4@L100-55+DSS, following the delayed treatment protocol. Representative images of spleens from each group are presented (n = 3 biologically independent animals per group).

**Figure S14. Representative H&E-stained images of colon sections within the delayed treatment model.**

Representative H&E-stained images and their corresponding locally magnified views of colon sections from the indicated experimental groups to highlight detailed histopathological changes. Black arrows indicate regions of inflammatory infiltration. Scale bars, 400 μm for the overview images and 100 μm for the locally magnified views.

**Figure S15. Intestinal retention kinetics of engineered strains in the delayed treatment model.**

Engineered bacteria were orally administered to mice following the delayed treatment protocol. To monitor bacterial accumulation during the intervention, fecal samples were collected daily prior to each administration (Days 7–11) and at the time of sacrifice (Day 12), and bacterial loads were quantified via plate colony counting. Fecal counts are expressed as Log_10_ CFUs. Data are presented as mean ± SEM (n = 5 biologically independent animals per group).

**Figure S16. Representative fluorescence images of ROS in mouse colon sections.**

Following the delayed treatment protocol, colon tissues were collected from mice in the indicated groups: PBS, DSS, Con+DSS, EB2+DSS, EB4+DSS, and EB4@L100-55+DSS. ROS production was evaluated using a specific fluorescence probe (red), and cell nuclei were counterstained with DAPI (blue). Representative merged images (DAPI/ROS) are presented to show the distribution of ROS within the colonic architecture. Scale bar denotes 200 μm.

**Figure S17. Myeloperoxidase (MPO) activity in colon tissues within the delayed treatment model.**

MPO activity was quantified in colon tissues harvested from mice in the indicated experimental groups, including PBS, DSS, Con+DSS, EB2+DSS, EB4+DSS, and EB4@L100-55+DSS. Data are presented as mean ± SEM (n = 5 biologically independent animals per group). Statistical significance was determined via one-way ANOVA followed by Tukey’s post-hoc test (****p < 0.0001; ns, not significant).

**Figure S18. Representative immunofluorescence images of tight junction proteins in mouse colon sections.**

Colon tissues were harvested from mice in the indicated experimental groups (PBS, DSS, Con+DSS, EB2+DSS, EB4+DSS, and EB4@L100-55+DSS) following the delayed treatment protocol. The expression and localization of tight junction proteins, including Claudin (red), Occludin (magenta), and ZO-1 (green), were evaluated via immunofluorescence staining. Cell nuclei were counterstained with DAPI (blue). Merged images are presented to illustrate the integrity of the intestinal mucosal barrier across different groups. Scale bar denotes 200 μm.

**Figure S19. Alpha diversity (Simpson index) of gut microbiota in the delayed treatment model.**

Microbial α-diversity in fecal samples was assessed via the Simpson index across the indicated experimental groups. Data are presented as mean ± SD (n = 5 biologically independent animals per group). Statistical significance was determined using a one-way ANOVA followed by Tukey’s post-hoc test (*p < 0.05).

**Figure S20. Beta diversity (PCA plot) of gut microbiota in the delayed treatment model.**

Microbial β-diversity was assessed via Principal Component Analysis (PCA) based on the fecal microbiota composition across the indicated groups. Variation explained by PC1 (9.73%) and PC2 (7.19%) is displayed on the axes. Individual samples are represented by points (n = 5 biologically independent animals per group), and ellipses denote 95% confidence intervals for each group.

**Figure S21. Ternary plots of gut microbiota composition in the delayed treatment model.**

Distributions of microbial taxa are presented at the (a) phylum and (b) genus levels across the indicated experimental groups. Ternary plots compare the relative abundances of taxa among EB4@L100-55+DSS, EB4+DSS, and either the DSS or PBS groups. Each circle denotes a specific taxon, with the diameter proportional to its mean relative abundance. The position of each circle relative to the vertices indicates the degree of enrichment in the respective groups.

**Figure S22. Multiple group comparative analysis of representative taxa at the genus level within the delayed treatment model.**

The mean proportions of *Lachnospiraceae*_NK4A136 (a), *Colidextribacter* (b), *Escherichia-Shigella* (c), and *Turicimonas* (d) were quantified in samples from mice in the indicated experimental groups, including PBS, DSS, Con+DSS, EB2+DSS, EB4+DSS, and EB4@L100-55+DSS. Data are presented as mean ± SEM (n = 5 biologically independent animals per group). Statistical significance was determined via one-way ANOVA followed by Tukey’s post-hoc test (*p < 0.05).

**Figure S23. Butanoic acid concentration in feces within the delayed treatment model.**

Butanoic acid levels were quantified in fecal samples collected from mice in the indicated experimental groups, including PBS, DSS, Con+DSS, EB2+DSS, EB4+DSS, and EB4@L100-55+DSS. Data are presented as mean ± SEM (n = 5 biologically independent animals per group). Statistical significance was determined via one-way ANOVA followed by Tukey’s post-hoc test (****p < 0.0001, *p < 0.05).

**Figure S24. Representative images of mouse spleens in the prophylactic treatment model.**

Spleens were harvested from mice across the indicated experimental groups, including PBS, DSS, Con+DSS, EB2+DSS, EB4+DSS, and EB4@L100-55+DSS, following the prophylactic treatment protocol. Representative images of spleens from each group are presented (n = 3 biologically independent animals per group).

**Figure S25. Representative H&E-stained images of colon sections within the prophylactic model.**

Representative H&E-stained images and their corresponding locally magnified views of colon sections from the indicated experimental groups to highlight detailed histopathological changes. Black arrows indicate regions of inflammatory infiltration. Scale bars, 400 μm for the overview images and 100 μm for the locally magnified views.

**Figure S26. Intestinal retention kinetics of engineered strains in the prophylactic treatment model.**

Engineered bacteria were orally administered to mice following the prophylactic treatment protocol. To monitor bacterial accumulation during the intervention, fecal samples were collected daily prior to each administration (Days 2–6) and at the time of sacrifice (Day 7), and bacterial loads were quantified via plate colony counting. Fecal counts are expressed as Log_10_ CFUs. Data are presented as the mean ± SEM (n = 5 biologically independent animals per group).

**Figure S27. Representative fluorescence images of ROS in mouse colon sections.**

Following the prophylactic treatment protocol, colon tissues were collected from mice in the indicated groups: PBS, DSS, Con+DSS, EB2+DSS, EB4+DSS, and EB4@L100-55+DSS. ROS production was evaluated using a specific fluorescence probe (red), and cell nuclei were counterstained with DAPI (blue). Representative merged images (DAPI/ROS) are presented to show the distribution of ROS within the colonic architecture. Scale bar denotes 200 μm.

**Figure S28. MPO activity in colon tissues within the prophylactic model.**

MPO activity was quantified in colon tissues harvested from mice in the indicated experimental groups (PBS, DSS, Con+DSS, EB2+DSS, EB4+DSS, and EB4@L100-55+DSS) following the prophylactic protocol. Data are presented as the mean ± SEM (n = 5 biologically independent animals per group). Statistical significance was determined via one-way ANOVA followed by Tukey’s post-hoc test (****p < 0.0001; ***p < 0.001; *p < 0.05).

**Figure S29. Representative immunofluorescence images of tight junction proteins in mouse colon sections.**

Colon tissues were harvested from mice in the indicated experimental groups (PBS, DSS, Con+DSS, EB2+DSS, EB4+DSS, and EB4@L100-55+DSS) following the prophylactic treatment protocol. The expression and localization of tight junction proteins, including Claudin (red), Occludin (magenta), and ZO-1 (green), were evaluated via immunofluorescence staining. Cell nuclei were counterstained with DAPI (blue). Merged images are presented to illustrate the integrity of the intestinal mucosal barrier across different groups. Scale bar denotes 200 μm.

**Figure S30. Alpha diversity (Simpson index) of gut microbiota in the prophylactic treatment model.**

16S rRNA sequencing was performed on fecal samples harvested from mice in the indicated experimental groups (PBS, DSS, Con+DSS, EB2+DSS, EB4+DSS, and EB4@L100-55+DSS). Microbial α-diversity was assessed via the Simpson index. Data are presented as mean ± SD (n = 5 biologically independent animals per group). Statistical significance was determined using a one-way ANOVA followed by Tukey’s post-hoc test.

**Figure S31. Beta diversity (PCA plot) of gut microbiota in the prophylactic treatment model.**

Microbial β-diversity was assessed via Principal Component Analysis (PCA) based on the fecal microbiota composition across the indicated groups. Variation explained by PC1 (12.84%) and PC2 (7.71%) is displayed on the axes. Individual samples are represented by points (n = 5 mice per group), and ellipses denote 95% confidence intervals for each group.

**Figure S32. Ternary plots of gut microbiota composition in the prophylactic treatment model.**

Distributions of microbial taxa are presented at the (a) phylum and (b) genus levels across the indicated experimental groups. Ternary plots compare the relative abundances of taxa among EB4@L100-55+DSS, EB4+DSS, and either the DSS or PBS groups. Each circle denotes a specific taxon, with the diameter proportional to its mean relative abundance. The position of each circle relative to the vertices indicates the degree of enrichment in the respective groups.

**Figure S33. Multiple group comparative analysis of representative taxa at the genus level within the prophylactic model.**

The mean proportions of *Lachnospiraceae*_NK4A136 (a), *Lactobacillus* (b), *Escherichia-Shigella* (c), and *Akkermansia* (d) were quantified in samples from mice in the indicated experimental groups, including PBS, DSS, Con+DSS, EB2+DSS, EB4+DSS, and EB4@L100-55+DSS. Data are presented as mean ± SEM (n = 5 biologically independent animals per group). Statistical significance was determined via one-way ANOVA followed by Tukey’s post-hoc test (**p < 0.01; *p < 0.05).

**Figure S34. Butanoic acid concentration in feces within the prophylactic treatment model.**

Butanoic acid levels were quantified in fecal samples collected from mice in the indicated experimental groups, including PBS, DSS, Con+DSS, EB2+DSS, EB4+DSS, and EB4@L100-55+DSS. Data are presented as mean ± SEM (n = 5 biologically independent animals per group). Statistical significance was determined via one-way ANOVA followed by Tukey’s post-hoc test (****p < 0.0001).

**Figure S35. Representative images of mouse colons in the delayed treatment model.**

Colons were harvested from mice across the indicated experimental groups, including PBS, DSS, Con+DSS, Con@L100-55+DSS, EB2+DSS, EB4(L-ara-)+DSS, EB4+DSS, EB4@L100-55+DSS, and 5-ASA+DSS, following the delayed treatment protocol. Representative images of colons from each group are presented. (n = 5 biologically independent animals per group).

**Figure S36. Representative images of mouse spleens in the delayed treatment model.**

Spleens were harvested from mice across the indicated experimental groups, including PBS, DSS, Con+DSS, Con@L100-55+DSS, EB2+DSS, EB4(L-ara-)+DSS, EB4+DSS, EB4@L100-55+DSS, and 5-ASA+DSS, following the delayed treatment protocol. Representative images of spleens from each group are presented. (n = 5 biologically independent animals per group).

**Figure S37. Representative H&E-stained images of mouse colon sections.**

Following the delayed treatment protocol, colon tissues were collected from mice in the indicated groups: PBS, DSS, Con+DSS, Con@L100-55+DSS, EB2+DSS, EB4(L-ara-)+DSS, EB4+DSS, EB4@L100-55+DSS, and 5-ASA+DSS. Histopathological changes were evaluated using H&E staining. Representative images are presented to show the histopathological changes within the colonic architecture. Black arrows indicate areas of inflammatory cell infiltration. Scale bars, 500 μm for the overview images and 200 μm for the locally magnified views.

**Figure S38. Biosafety evaluation of the final therapeutic formulation EB4@L100-55 in healthy and colitic mice.**

(a) Safety evaluation via body weight monitoring in healthy mice. Healthy mice were orally administered 1 × 10⁹ CFU of EB4@L100-55 or an equal volume of PBS buffer once daily for seven consecutive days. Body weight was monitored daily and is expressed as a percentage of the initial weight on Day 0. (b) Representative H&E-stained images of major organs (heart, liver, spleen, lung, and kidney) and colonic tissues from healthy mice following the complete treatment regimen. (c) Representative H&E-stained images of extra-intestinal major organs and colonic tissues from DSS-induced colitic mice following the delayed treatment regimen with PBS or EB4@L100-55. Data are presented as mean ± SEM (n = 5 biologically independent animals per group). Scale bars, 200 μm. Statistical significance between groups over time was determined using a two-way ANOVA followed by Sidak's multiple comparisons test.

**Figure S39. Self-limiting clearance kinetics of the engineered strains in the delayed-treatment colitis model.**

Mice were treated with 2.5% DSS from Day 0 to Day 7 to induce acute colitis, followed by daily oral administration of the indicated strains (1 × 10^9^ CFU) from Day 7 to Day 11. Fecal samples were collected daily starting from Day 7, prior to the initial gavage, until the engineered bacteria were entirely cleared across all groups. (a) Overall fecal bacterial abundance profiles during the treatment and post-treatment clearance periods. Bacterial loads were quantified by plate colony counting on selective agar and are expressed as Log_10_ CFU/g of feces. (b) Single-exponential decay fitting of the clearance-phase data using raw CFU/g values, with the plateau constrained to 0. Experimental Days 12–20 were re-coded as Days 0–8 of the clearance phase, with Day 0 denoting the first fecal sampling point after the final bacterial administration. Symbols and error bars represent the mean ± SEM (n = 5 biologically independent animals per group), and solid lines represent the best-fit curves. Adhesion-enhanced strains and non-adhesive or uninduced controls are displayed in separate subpanels with different y-axis ranges to improve visualization. (c) Best-fit clearance rate constants (*k*, d^-1^) and half-lives (*T*_1/2_, d), together with their profile-likelihood 95% confidence intervals.

**Figure S40. Modular construction strategy of engineered EcN strains.**

The schematic illustrates the stepwise assembly of functional modules into the pET-INPN-tSpaC base plasmid. The mGFP reporter was first integrated into the INPN-tSpaC frame, followed sequentially by the CAT-SOD-GPx tripartite fusion system (TFS)-based antioxidant module and the TFF3 secretion module. EcN strains harboring these progressively constructed plasmids are designated as EcN-EB1 through EcN-EB4. The final construct represents the complete therapeutic platform (EcN-EB4).

**Table S1. Strains used in this study.**

| **Strains** | **Characteristics** | **Source** |
| --- | --- | --- |
| *Escherichia coli* DH10B | Cloning host | BeNa Culture Collection, China |
| *Escherichia coli* BL21(DE3) | Protein expression host | Our laboratory |
| *Escherichia coli* Nissle 1917 | Human commensal *E. coli* isolate | BeNa Culture Collection, China |
| *Lactobacillus casei* ATCC393 | Target gene source | BeNa Culture Collection, China |
| *Lactococcus lactis* NZ9000 | Target gene source | BeNa Culture Collection, China |
| *Bacillus subtilis* 168 | Target gene source | Generously provided by Zhemin Zhou’s Lab, Jiangnan University |
| EcN-Con | EcN harboring pET-mGFP | This study |
| EcN-EB1 | EcN harboring pET-araBAD-tSpaC | This study |
| EcN-EB2 | EcN harboring pET-tSpaC-mGFP | This study |
| EcN-EB3 | EcN harboring pET-tSpaC-mGFP-TFS | This study |
| EcN-EB4 | EcN harboring pET-tSpaC-mGFP-TFS-TFF3 | This study |
| EB4@L100-55 | EcN-EB4 microencapsulated with pH-responsive Eudragit L100-55 | This study |
| EcN-DE(Bs) | EcN harboring pBAD-DE(Bs); expresses CAT-SOD(Bs) dual-enzyme fusion | This study |
| EcN-DE(Ll) | EcN harboring pBAD-DE(Ll); expresses CAT-SOD(Ll) dual-enzyme fusion | This study |
| EcN-TFS(Bs) | EcN harboring pBAD-TFS(Bs); expresses CAT-SOD(Bs)-GPx tripartite fusion construct | This study |
| EcN-TFS(Ll) | EcN harboring pBAD-TFS(Ll); expresses CAT-SOD(Ll)-GPx tripartite fusion construct | This study |
| EcN-ykgMO-mGFP | EcN harboring pET-ykgMO-mGFP | This study |
| BL21(DE3)-S100A8A9 | BL21(DE3) harboring pRSFDuet-S100A8A9 | This study |

**Table S2. The primers used in this study.**

| **Plasmid** | **Primer** | **Sequence (5’**–**3’)** |
| --- | --- | --- |
| pET22b-mGFP | mGFP-F | cgatcttgacagctagctcagtcctaggtataatgctagctac |
|  | mGFP-R | cctcctttcagcgcagaaaggcccacccgaag |
|  | 22b-F | ctgaaaggaggaactatatccggattggcgaatg |
|  | 22b-R | tagctgtcaagatcgagatctcgatcctctacgccggac |
| pET22b-SpaC | Para-F | ttatgacaacttgacggctacatcattcactttttct |
|  | Para-R | atgtatatctccttcttatacttaactaatatactaagatgatggagaaacagtagagagttgc |
|  | INPN-SpaC-F | gaaggagatatacatatgaaatacctgctgccgaccgc |
|  | INPN-SpaC-R | agatctcaaaaaacccctcaagacccgtttagagg |
|  | ConVec-F | ggttttttgagatctttgacagctagctcagtcctaggt |
|  | ConVec-R | cgtcaagttgtcataagatcgagatctcgatcctctacgccg |
| pBAD-DE(Bs) | BsSOD-F | gcagcagcaaaagaggcagcagcaaaggctatggcttacgaacttccagaattaccttatgcgtac |
|  | BsSOD-R | agccggttattttgcttcgctgtatagacgagccacttc |
|  | BsVec-F | gcaaaataaccggctgctaacaaagcccgaaag |
|  | SODVec-R | ctcttttgctgctgcctccttagccgctgcttctgcaccttggtggttgtgtaatctaggatcacc |
| pBAD-DE(Ll) | LlSOD-F | gcagcagcaaaagaggcagcagcaaaggctatggcatttacattacctgaacttccatatgcg |
|  | LlSOD-R | ttattttgccttagcgtaaagttcattgactttatcccag |
|  | LlVec-F | gctaaggcaaaataaccggctgctaacaaagcccga |
| pBAD-TFS(Bs) | GPX-F | ggaggcggcggcagcggaggtggcgggagcatgtcaatttatgatttcgatgcaactttggaagatgg |
|  | GPX-R | ttaagcaagaacctgtttcaacgccgg |
|  | TFSVec-F | caggttcttgcttaaccggctgctaacaaagcccg |
|  | TFSVec-Bs-R | gctgccgccgcctccagagccgcctccaccttttgcttcgctgtatagacgagccacttc |
| pBAD-TFS(Ll) | TFSVec-Ll-R | gctgccgccgcctccagagccgcctccaccttttgccttagcgtaaagttcattgactttatcccag |
| pET22b-SpaC-TFS | TFS-F | gagcatgtatctttacggttatgttataacataacaggtaaaaatggactacaaagaccatgacggtgattataaagatcatg |
|  | TFS-R | caaaaaacccctcaagacccgtttagaggc |
|  | SpaCVec-F | ttgaggggttttttgctgaaaggaggaactatatccggattggcgaatg |
|  | SpaCVec-R | taaagatacatgctcatccggatcatttcaacacccaatcactgtgactatttgtggcgcagaaaggcccacccgaag |
| pET28a-yebF-TFF3 | yebF-F | tgttataacataacaggtaaaaatgaaaaaaagaggggcgtttttagggctg |
|  | yebF-R | ttccatctcgagacgccgctgatattccgcca |
|  | TFF3Vec-F | cgtctcgagatggaagaatatgtgggcctgagcg |
|  | TFF3Vec-R | tgttatgttataacataaccgtaaagatacatgctcatccggatc |
| pET22b-SpaC-TFS-TFF3 | yebF-TFF3-F | tggcgccacaaatagtcacagtgattgggtgttgaaatgatcc |
|  | yebF-TFF3-R | caataggccgaaatcggcaaaatcccttataaatc |
|  | S-TFS-Vec-F | ccgatttcggcctattggttaaaaaatgagctg |
|  | S-TFS-Vec-R | gactatttgtggcgccaatccggatatagttcctcc |
| pET22b-ykgMO-mGFP | ykgMO-F | ggatgagcatgtatctttacggttatgttataacataacaggtaaaaatgatggtttctaagggcgaagaagacaacatg |
|  | ykgMO-R | gatacatgctcatccggatcatttcaacacccaatcactgtgactatttgtggagatctcgatcctctacgccggacg |

**Table S3. Disease activity index (DAI) parameters and their associated scoring schemes.**

| **Score** | **Weight loss (%)** | **Stool consistency** | **Blood in stool** |
| --- | --- | --- | --- |
| 0 | None | Normal | Normal |
| 1 | 1–5 | Slightly loose stool | Small presence of blood |
| 2 | 5–10 | Loose stool | Significant presence of blood |
| 3 | 10–15 | Diarrhea | Gross blood |
| 4 | ＞15 |  |  |
